# Cultivation of novel *Atribacterota* from oil well provides new insight into their diversity, ecology, and evolution in anoxic, carbon-rich environments

**DOI:** 10.1186/s40168-024-01836-7

**Published:** 2024-07-06

**Authors:** Jian-Yu Jiao, Shi-Chun Ma, Nimaichand Salam, Zhuo Zhou, Zheng-Han Lian, Li Fu, Ying Chen, Cheng-Hui Peng, Yu-Ting OuYang, Hui Fan, Ling Li, Yue Yi, Jing-Yi Zhang, Jing-Yuan Wang, Lan Liu, Lei Gao, Aharon Oren, Tanja Woyke, Jeremy A. Dodsworth, Brian P. Hedlund, Wen-Jun Li, Lei Cheng

**Affiliations:** 1 State Key Laboratory of Biocontrol, Guangdong Provincial Key Laboratory of Plant Resources and Southern Marine Science and Engineering Guangdong Laboratory (Zhuhai), School of Life Sciences, Sun Yat-Sen University, Guangzhou, 510275 People’s Republic of China; 2 Key Laboratory of Development and Application of Rural Renewable Energy, Biogas Institute of Ministry of Agriculture and Rural Affairs, Chengdu, 610000 People’s Republic of China; 3 National Agri-Food Biotechnology Institute, Sector-81 (Knowledge City), Mohali, 140306 Punjab India; 4 Department of Plant and Environmental Sciences, The Alexander Silberman Institute of Life Sciences, The Edmond J. Safra Campus, The Hebrew University of Jerusalem, Jerusalem, 9190401 Israel; 5 DOE Joint Genome Institute, Lawrence Berkeley National Laboratory, Berkeley, CA USA; 6 University of California Merced, Life and Environmental Sciences, Merced, CA USA; 7 California State University, San Bernardino, CA USA; 8 School of Life Sciences, University of Nevada Las Vegas, Las Vegas, NV 89154 USA; 9 Nevada Institute of Personalized Medicine, University of Nevada Las Vegas, Las Vegas, NV 89154 USA; 10 State Key Laboratory of Desert and Oasis Ecology, Key Laboratory of Ecological Safety and Sustainable Development in Arid Lands, Xinjiang Institute of Ecology and Geography, Chinese Academy of Sciences, Urumqi, 830011 People’s Republic of China

**Keywords:** *Atribacterota*, *Atribacteria*, *Phoenicimicrobiia*, Pure culture, Enrichment, Wood-Ljungdahl pathway, Reductive glycine pathway, Oil reservoir, Carbohydrate fermentation, Hydrocarbon degradation

## Abstract

**Background:**

The *Atribacterota* are widely distributed in the subsurface biosphere. Recently, the first *Atribacterota* isolate was described and the number of *Atribacterota* genome sequences retrieved from environmental samples has increased significantly; however, their diversity, physiology, ecology, and evolution remain poorly understood.

**Results:**

We report the isolation of the second member of *Atribacterota*, *Thermatribacter velox* gen. nov., sp. nov., within a new family *Thermatribacteraceae* fam. nov., and the short-term laboratory cultivation of a member of the JS1 lineage, *Phoenicimicrobium oleiphilum* HX-OS.bin.34^TS^, both from a terrestrial oil reservoir. Physiological and metatranscriptomics analyses showed that *Thermatribacter velox* B11^T^ and *Phoenicimicrobium oleiphilum* HX-OS.bin.34^TS^ ferment sugars and *n*-alkanes, respectively, producing H_2_, CO_2,_ and acetate as common products. Comparative genomics showed that all members of the *Atribacterota* lack a complete Wood-Ljungdahl Pathway (WLP), but that the Reductive Glycine Pathway (RGP) is widespread, indicating that the RGP, rather than WLP, is a central hub in *Atribacterota* metabolism. Ancestral character state reconstructions and phylogenetic analyses showed that key genes encoding the RGP (*fdhA*, *fhs*, *folD*, *glyA*, *gcvT*, *gcvPAB*, *pdhD*) and other central functions were gained independently in the two classes, *Atribacteria* (OP9) and *Phoenicimicrobiia* (JS1), after which they were inherited vertically; these genes included fumarate-adding enzymes (*faeA*; *Phoenicimicrobiia* only), the CODH/ACS complex (*acsABCDE*), and diverse hydrogenases (NiFe group 3b, 4b and FeFe group A3, C). Finally, we present genome-resolved community metabolic models showing the central roles of *Atribacteria* (OP9) and *Phoenicimicrobiia* (JS1) in acetate- and hydrocarbon-rich environments.

**Conclusion:**

Our findings expand the knowledge of the diversity, physiology, ecology, and evolution of the phylum *Atribacterota*. This study is a starting point for promoting more incisive studies of their syntrophic biology and may guide the rational design of strategies to cultivate them in the laboratory.

Video Abstract

**Supplementary Information:**

The online version contains supplementary material available at 10.1186/s40168-024-01836-7.

## Background

Members of the phylum *Atribacterota* are found in a broad range of anoxic environments, including oil reservoirs [1] and produced water [2], hot springs [3], marine sediments [4], and methane hydrates [5]. Currently, only one species, *Atribacter laminatus* RT761^T^, has been isolated and characterized [2]. *A. laminatus*, a glucose fermenter, produced H_2_, acetate, and CO_2_ as fermentation products and was stimulated by co-cultivation with a hydrogenotrophic methanogen [2]. This metabolism, along with the previous enrichment of two *Candidatus* Caldatribacterium species on lignocellulose suggested widespread sugar fermentations within the class *Atribacteria*, known informally as OP9 [6]. However, this function is not well understood due to poor cultivability and a paucity of high-quality genomes of *Atribacterota*. Interpretations of additional single-amplified genomes (SAGs) and metagenome-assembled genomes (MAGs) and their environmental origins have also hinted at anaerobic hydrocarbon fermentation and either syntrophic acetate oxidation or homoacetogenesis via the Wood-Ljungdahl pathway (WLP) [1–5, 7]. Although these studies provide valuable insights into these organisms, the presence of only a single pure culture and the scarcity of experimental studies targeting *Atribacterota* obscure a more comprehensive understanding of the phylum’s nature.

In this study, we describe the isolation of a second pure culture of *Atribacterota*, herein named *Thermatribacter velox* B11^T^, and hydrocarbon-degrading enrichments containing a member of the JS1 class, herein named *Phoenicimicrobium oleiphilum* HX-OS.bin.34^TS^. We also probe the core metabolism of *Atribacterota*, which revealed the importance of the reductive glycine pathway (RGP), and not the WLP, in the core metabolism of *Atribacterota*. We suggest a central metabolic core of the RGP and diverse hydrogenases, with the variable presence of glycoside hydrolases, fumarate-adding enzymes (Fae), CO dehydrogenase/acetyl-CoA synthase (AcsABCDE), and formate dehydrogenase (FdhAB) endow *Atribacterota* with different roles in syntrophic networks in a variety of anoxic, carbon-rich environments.

## Methods

### Sample collection and acetate enrichment

Crude oil sludge samples were collected from the Haoxian Oil Reservoir (HX-OS) that originated between 1000 and 2000 m deep with a temperature range of 60–80 °C [8], which is part of the Shengli Oilfield located in Shandong Province, China (E 118°31′30′′, N 37°24′00′′) in April 2017. Anaerobic enrichment cultures were set up (Figure S1), initially using a pre-reduced medium (PRM) at 75 °C as described previously [8]. Following incubation for 300 days, 10 ml of culture was inoculated into 20 ml of PRM supplemented with 10 mM acetate (culture HX-AS) and incubated at 75 °C. After 100 days, culture HX-AS was subcultured and transferred at an interval of approximately 30 days by inoculating 20 ml cultures into 600 ml serum bottles containing 20 ml PRM supplemented with 10 mM acetate to enrich for an acetate-degrading methanogenic consortium (Figure S2). Acetate consumption and methane production were detected using high-performance liquid chromatography (HPLC, LC 1200, Agilent) and gas chromatography (GC 7820A, Agilent) as described previously [8].

### Isolation and complete genome sequencing of B11

Previous genomic studies have suggested syntrophic acetate oxidation in *Atribacterota* [4, 9, 10]. Therefore, we targeted acetate-degrading *Atribacterota* from the enrichment culture HX-AS [11] using the anaerobic roll-tube method to obtain colonies [12, 13] by using a low-salt (LS) medium supplemented with 10 mM sodium acetate. The LS medium contained (per liter) 9 g NaCl, 3 g MgCl_2_·6H_2_O, 0.15 g CaCl_2_·2H_2_O, 0.3 g NH_4_Cl, 0.2 g KH_2_PO_4_, 0.5 g KCl, 7.16 g HEPES, 2 ml trace elements solution, 2 ml vitamins solution [14], 2.52 g NaHCO_3_, 0.5 g Na_2_S·9H_2_O, and 0.5 ml resazurin solution (1 g/L). Ten millimeter sodium acetate and 15 g/L Gelzan^TM^ CM were used as substrate and solidifying agents, respectively. The medium was prepared under a gas mixture of N_2_:CO_2_ (80:20) using modified Hungate anaerobic techniques [15]. Vitamins, NaHCO_3,_ and Na_2_S·9H_2_O were autoclaved separately from sterile anaerobic solutions at 121 °C for 30 min and added to the medium. The final pH of the medium was adjusted to 6.8–7.0. Approximately 0.5 ml enrichment cultures were inoculated into Hungate tubes containing LS medium supplemented with 10 mM sodium acetate after a series of dilutions (10^−1^ to 10^−8^). All tubes were incubated at 75 °C. Following incubation for 2 to 4 weeks, single colonies were picked and transferred into the liquid isolation medium. The purified isolates were identified by calculating sequence similarities of partial 16S rRNA gene sequences using NCBI Blast. DNA extraction, 16S rRNA gene amplification, and sequencing were performed according to methods described previously [16] using the primer set 27F/1492R [16, 17]. Subsequently, the colonies identified as members of the *Atribacteria* were purified by repeated restreaking onto roll-tubes. A single colony designated as B11^T^ was obtained. The purity of strain B11^T^ was confirmed by microscopic observation and repeated sequencing of the 16S rRNA gene (OQ519918). As strain B11^T^ grew slowly in LS medium with acetate as the sole carbon source, a nutrient-rich LS medium containing 10 mM glucose and 0.5 g/L yeast extract was used for subsequent culturing. For whole genome sequencing, genomic DNA of strain B11^T^ was extracted from cells harvested at the exponential phase using the ZYMO HMW gDNA kit (ZYMO, California, USA) according to the manufacturer’s instructions. The whole genome was sequenced using Illumina NovaSeq PE150 at the Beijing Novogene Bioinformatics Technology Co., Ltd. Genome assembly was performed using SOAPdenovo (version 2.04) [18], SPAdes [19] and ABySS [20], followed by CISA [21] to integrate the assemblies. Gaps were filled with gapclose (version 1.12), thereby generating a closed, circular genome (CP121689). Default parameters were used for all the assembly and gap closure steps.

### Microscopic observation

Cultures of strain B11^T^ grown in the nutrient-rich LS medium at 75 °C for 48 h were used to determine cell morphology and structure. Gram staining was performed using a Gram-staining kit (Solarbio, China) and was observed with microscopy (Eclipse 80i, Nikon). Cell shape and size were observed with scanning electron microscopy (EVO18, Zeiss). Samples washed with sodium phosphate buffer (PBS) (0.01 M, pH 7.2) were fixed with 2.5% glutaraldehyde at 4 °C for 12 h, washed thrice with PBS, and dehydrated in a series of ethanol and with tert-butyl alcohol for 15 min each. The sample was then kept at −80 °C for 12 h, freeze-dried in a lyophilizer (FreeZone^®^6, Labconco), and finally coated with gold before observation. Cell structures were examined by transmission electron microscopes (TEMs) using negative staining and ultramicrotomy. To prepare for negative staining, 2 μl of active cultures were dropped on formvar film-coated copper grids (200 mesh) and dried at room temperature. Samples were stained for 1 min with neutral 1% (w/v) phosphotungstic acid solution, excess reagent washed with ultrapure water, air-dried, and observed in a field emission transmission electron microscopy (Tecnai G2 F20 S-TWIN, FEI). To prepare thin slices for TEM observation, cells were prefixed with 3% glutaraldehyde at 4 °C for 12 h and then postfixed in 1% osmium tetroxide at 4 °C for 2 h, dehydrated in a gradient series of acetone, infiltrated and embedded in Epon 812. The semi-thin sections were stained with methylene blue. Ultrathin sections were cut with an ultramicrotome (EM UC7, Leica), and stained with uranyl acetate and lead citrate. Sections were examined with a TEM (JEM-1400-FLASH, JEOL).

### Physiological characterization

Physiological tests for strain B11^T^ including temperature, pH, and potential fermentation substrates were performed in the nutrient-rich LS medium containing 10 mM glucose and 0.5 g/L yeast extract unless otherwise indicated. The NaCl concentration range was tested in a NaCl-free medium. Substrate utilization was determined in LS medium supplemented with 0.2 g/L yeast extract and potential fermentation substrates; LS medium supplemented with 0.2 g/L yeast extract was used as control. Growth of strain B11^T^ was determined by monitoring the optical density at 600 nm (OD_600_) with a cell density meter (Ultrospec 10, biochrom). Metabolites in the gaseous phase (hydrogen and carbon dioxide) were measured with gas chromatography (GC) equipped with a thermal conductivity detector (GC 2010, Shimadzu) and a Porapak Q column. Ethanol in the liquid phase was measured using a GC (GC 7890A, Agilent) equipped with a flame ionization detector and DB-WAX column (length, 30 m; inner diameter, 0.32 mm), while lactate, formate, acetate, propionate, butyrate, and iso-valerate were measured using a HPLC (LC 1200, Agilent) equipped with Aminex HPX-87H column (Bio-Rad).

Oxygen tolerance was tested in Hungate anaerobic tubes containing the anaerobic nutrient-rich LS medium which contains 10 mM glucose and 0.5 g/L yeast extract, but without reducing agents (Na_2_S or cysteine). Different volumes of oxygen were added into the headspace of the tube (0, 1, 2, and 5%). OD_600_ was measured to determine the growth of the isolate.

### Chemotaxonomic analyses

Cells incubated in the nutrient-rich LS medium at 70 °C for 48 h were used to analyze cellular fatty acids, polar lipids, and respiratory quinones. The methyl esters of fatty acids were prepared and extracted from freeze-dried biomass according to the protocol described by Eder [22]. The esterified fatty acids were separated with a gas chromatograph-mass spectrometer (8860 GC system with 5977B GC/MSD, Agilent), and identified with Sherlock Microbial Identification System (MIDI Inc, Newark). Polar lipids and respiratory quinones were extracted following a previously published protocol [23]. The purified respiratory quinones were analyzed with HPLC (1260 Infinity II, Agilent) [24].

### DNA/RNA extraction and next generation sequencing

DNA for metagenomic sequencing was collected from 10 to 15 g of oil sludge (HX-OS) or from culture HX-AS, and DNA was extracted using bead-beating methods [25]. DNA libraries were generated by Novogene using a TruSeq DNA PCR-Free kit for Illumina (CA, USA) according to the manufacturer’s instructions.

For metatranscriptomic sequencing, cells were collected from about 15–20 ml of the sample from the oil reservoir (HX-OS) by centrifugation (17,000×*g*, 5 min, 4 °C; Beckman Coulter), transferred immediately to liquid nitrogen, and stored at −80 °C. Cells were lysed by using the bead-beating method (FastPrep-24, MP), and total RNA was extracted using an acid phenol:chloroform:isoamyl alcohol method [26]. Quality checking, genomic DNA digestion, ribosomal RNA removal, cDNA synthesis, and library construction were performed by Novogene. Metagenomic and metatranscriptomic sequencing were both performed using the NovaSeq 6000 instrument with PE150 at Novogene.

### Metagenomic and metatranscriptomic analyses

The raw metagenomic reads were quality filtered by using fastp [27] with the parameters (-q 20 -u 20 -e 20 -l 50). High-quality reads were *de novo* assembled using SPAdes [19] with parameters --meta -k 21,33,55,77,99. Genome binning was performed by using MetaBAT [28] as previously described [29], based on scaffolds with lengths > 2.5 kbp. The completeness and contamination of each MAG were calculated using CheckM [30]. MAGs with estimated completeness < 50% or contamination > 10% were discarded from this study as low-quality MAGs per MIMAG standards [31]. Medium- and high-quality MAGs were grouped into species clusters based on 95% average nucleotide identity (ANI) values determined by pyANI [32]. The genomes with the highest quality in each species cluster were selected as cluster representatives. A genome database comprised of the 33 representative genomes was then constructed and used for genome annotation and community function reconstruction. The Genome Taxonomy Database (GTDB)-Tk [33] was used to assign a taxonomy to the representative genomes by using relative evolutionary divergence (RED). The iRep (index of replication) [34] analysis was used to estimate the replication rate of each species in situ. Briefly, high-quality reads from each metagenome were mapped to the representative genomes by using Bowtie2 [35], and the read mapping data was then used for calculating the iRep values with default parameters. The relative abundance of taxa in HX-OS and HX-AS was examined by Kaiju software [36] with high-quality reads. Raw metatranscriptomic data were pre-processed as described for metagenomic data. The rRNA sequences from the resulting high-quality reads were removed by SortMeRNA [37] (--fastx --sam --aligned --other --paired_in --out2 -e 5 --de_novo_otu --otu_map). Finally, the filtered metatranscriptomic reads were mapped to the *Atribacterota* genes by BBmap v.38.85 with a sequence similarity threshold of 95% (http://sourceforge.net/projects/bbmap/).

### Phylogenetic analysis

A set of 48 *Atribacterota* genomes (Table S1) included new genomes from this study and high- and medium-quality species references from the Genome Taxonomy Database (completeness > 80% and contamination < 5%). The 16 ribosomal proteins were extracted and identified by using AMPHORA2 [38]. All marker proteins were aligned using the MUSCLE program [39] with 100 iterations. The TrimAL program [40] (-gt 0.95 -cons 50 -htmlout) was used to eliminate the poorly aligned regions, and finally, 2393 amino acid positions were kept for further analysis. The alignments were concatenated by using the Perl script (https://github.com/nylander/catfasta2phyml). A maximum-likelihood phylogeny was calculated by IQ-Tree [41] with parameters (-alrt 1000 -bb 1000 -nt AUTO). The best-fit model (LG+F+R5) is well supported by the Bayesian Information Criterion (BIC).

Datasets of genes of interest were derived from previous studies [1, 29, 42]. Maximum-likelihood phylogenies of concatenated protein sequences (*acsAB*, *acsABC*, and *acsDABCE*) were performed as described in previous studies, respectively [29, 42]. The proteins of each subunit were aligned using MUSCLE with 100 iterations [39]. The alignments were concatenated by using a Perl script with default parameters (https://github.com/nylander/catfasta2phyml). IQ-Tree [41] was used for phylogenetic inference with parameters (-alrt 1000 -bb 1000 -nt AUTO). The best-fit models for the phylogenetic trees of concatenated protein sequences (*acsAB*, *acsABC*, and *acsDABCE*) were LG+R7, LG+F+R8, and LG+F+R8, respectively.

The MrBayes [43] was used to construct the Bayesian tree with parameters (ngen = 1,000,000 Nruns = 2 Nchains = 4 diagnfreq = 1000 relburnin = yes burninfrac = 0.25 samplefreq=100 printfreq = 100) using the multiple sequence alignment of the concatenated ribosomal proteins. The results of the standard deviation of split frequencies (< 0.01), the potential scale reduction factor (PSRF = 1), and the effective sample size (ESS > 100) made the Bayesian tree highly reliable. The time-calibrated phylogenetic tree was further inferred by using R with the ape package. All trees were visualized and annotated by using iTOL.

### Comparative genomic analysis

The protein-coding sequences (CDSs) of each genome were predicted by using Prodigal [44] with the parameter “-p single”, and the CDSs were then annotated against egg-NOG and KEGG databases by using DIAMOND [45] with E-values < 1e-10. The predicted CDSs were also uploaded to the KEGG Automatic Annotation Server [46] with “for prokaryotes” and “bidirectional best hit” options for further function confirmation and metabolic pathway reconstruction. Average amino acid identity (AAI) values of each pair of genomes were calculated as described earlier [29]. The orthologous protein sequences were identified based on reciprocal best BLAST hits, and the mean identity of all orthologs was calculated as the AAI value. The 95% ANI value was used as the threshold for defining species [47]. The rRNAs and tRNAs were predicted by using RNAmmer [48] and tRNAscan-SE [49], respectively. Clusters of orthologous genes were generated by KEGG based on representative genomes. The evolutionary history of *Atribacterota* was inferred by using COUNT [50] as previously described [29, 51, 52]. Briefly, the gain-loss-duplication model with the Poisson family size distribution at the root was used for the calculation of the rate models. The rate variation across families was evenly distributed into four gamma categories: edge length, loss rate, gain rate, and duplication rate, each set at a 1:1:1:1 ratio. Convergence criteria were established at 100 rounds for maximum number of optimization rounds, with a 0.1 convergence threshold on the likelihood. Ancestral inference and family dynamics analyses were performed with the option Dollo parsimony.

### Laboratory enrichments of *Phoenicimicrobium oleiphilum* and metagenomic/metatranscriptomic analysis

Cultivation strategies were guided by multi-omics data [25] to test the degradation of *n*-alkanes in *Atribacterota*. The laboratory enrichments of oil sludge were performed in anoxic PRM at 55 °C with a variety of substrates, including 1 ml/L hexadecane, 2 ml/L eicosane, 0.6 g/L triacontane, and additional 200 g/L oil sludge, respectively. After 1 month of cultivation, 15–20 ml of each enrichment culture was collected, and DNA/RNA was extracted. Metagenomic/metatranscriptomic sequencing and analysis were performed as described above.

## Results and discussion

### Development of a long-term acetate-oxidizing culture containing *Atribacterota* from a crude oil reservoir

**Fig. 1:**
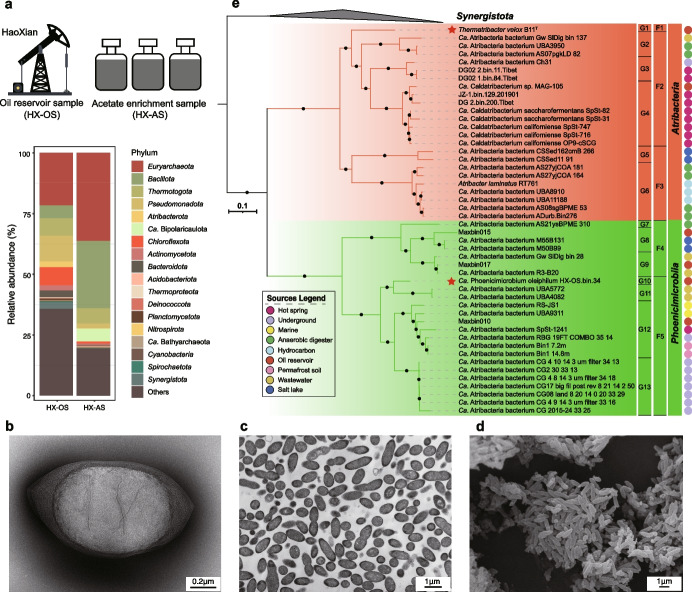
Overview of *Atribacterota* in the hydrocarbon- and acetate-enriched environments. **a** The community structures of oil reservoir and acetate enrichment samples that were examined by Kaiju software [36] with high-quality reads. **b**, **c** Transmission electron microscope (TEM) observations of *Thermatribacter velox* B11^T^. The intracellular membrane is visible, as described in *Atribacterota* previously [2]. **d** Scanning electron microscope (SEM) observation of ultrathin sections of *Thermatribacter velox* B11^T^. Cells of strain B11 were fusiform rod or ovoid shape with 0.4–0.5 μm in width and 0.6–1.9 μm in length. **e** Phylogenetic analysis of *Atribacterota*. F1: *Thermatribacteraceae* fam. nov. (G1: *Thermatribacter* gen. nov.); F2: *Caldatribacteriaceae* (G2: *Sordiicultor* gen. nov., G3: *Aquicavum* gen. nov., G4: *Caldatribacterium*); F3: *Atribacteraceae* (G5: *Nitricultor* gen. nov., G6: *Atribacter*); F4: *Stramentimicrobiaceae* fam. nov. (G7: *Stramentimicrobium* gen. nov., G8: *Oleincola* gen. nov., G9: *Oleihabitans* gen. nov.); F5: *Phoenicimicrobiiaceae* fam. nov. (G10: *Phoenicimicrobium* gen. nov., G11: *Immundihabitans* gen. nov., G12: *Sediminicultor* gen. nov., G13: *Infernicultor* gen. nov.). The maximum-likelihood phylogenetic tree was constructed using the concatenated alignment of 16 ribosomal proteins identified by using AMPHORA2 [38, 65]. All marker genes were aligned using the MUSCLE program [39] with 100 iterations, respectively. The TrimAL software [40] was used to eliminate the poorly aligned regions, and finally, 2393 amino acid positions were kept for further analysis. The alignments were concatenated and then used for calculating a maximum-likelihood phylogeny by IQ-Tree [41]. The best-fit model LG+F+R5 is well supported by the Bayesian Information Criterion. Bootstrap values were based on 1000 replicates and nodes with percentages > 80% were indicated as black circles. The red circles listed on the right illustrate the genome sources. The lineages with red stars showed the phylogenetic placement of novel lineages from this study

Oil sludge (HX-OS) that originated from 1000 and 2000 m deep within the Shengli Oilfield, China, was initially collected to study anaerobic acetate oxidation. Anaerobic enrichment cultures were continuously passaged at 75 ºC with 10 mM acetate as the sole source of carbon for nearly 4 years (culture HX-AS; Figure S1), after which amendments of 3 mM acetate were consumed reproducibly within 30 days, with the concomitant production of ~ 2.5 mM CH_4_ (Figure S2), implying acetoclastic methanogenesis or syntrophic acetate oxidation. To identify the microbial community members within the HX-AS enrichment culture and the original sample from the Haoxian Oil Reservoir (HX-OS), metagenomic assemblies were taxonomically assigned (Fig. 1a; Table S2), binned, and annotated, resulting in 33 representative high-quality MAGs based on 95% average nucleotide identity (ANI) (Table S3). The predominant archaea belonged to the *Euryarchaeota* (GTDB_id: p__Halobacteriota and p__Methanobacteriota) and contained *mcrABCDG* genes, suggesting they are involved in methane metabolism [53]. The predominant bacteria belonged to the *Bacillota* (*Firmicutes*), particularly classes *Moorelia* and *Thermoanaerobacteria*. These MAGs encode the Wood-Ljungdahl pathway, which is consistent with their potential roles in syntrophic acetate oxidation in the acetate-enriched culture [54] (Table S3).

Two MAGs (HX-AS.bin.3 and HX-OS.bin.34) were assigned to the classes *Atribacteria* (OP9) and JS1 within the phylum *Atribacterota* according to the GTDB-Tk [33]. The presence of HX-AS.bin.3, a member of class *Atribacteria*, in the long-term acetate-amended enrichment (0.31 % in HX-AS; Table S3) is consistent with a previously proposed role of some *Atribacterota* in syntrophic acetate oxidation [1–5, 7]. However, genes coding for the Wood-Ljungdahl pathway (*acsABCED*) were not detected in HX-OS.bin.34 and HX-AS.bin.3, which obscured their functions in these environments. The detection of *faeABCDEFJ* genes in HX-OS.bin.34 is consistent with the inferred genomic ability of some members of the JS1 group to ferment short-chain *n*-alkanes into fatty acids [1], and aligned with the relatively high abundance of this species in the oil reservoir sample (2.33% in HX-OS; Table S3).

### Laboratory isolation of the second member of *Atribacterota* from an acetate-amended enrichment

To evaluate the potential role of HX-AS.bin.3 in the acetate-amended enrichment, anaerobic isolation was performed by using the acetate enrichment culture HX-AS as the inoculum. The isolate B11^T^ was obtained on a low salt (LS) medium supplemented with 10 mM acetate and represents the second pure culture in the phylum *Atribacterota* (Fig. 1b–d; Supplementary information; Table S4). Although strain B11^T^ was isolated on LS medium supplemented with acetate as the only source of carbon, B11^T^ could not be maintained on the same medium. Correspondingly, the key genes encoding the CO dehydrogenase/acetyl-CoA synthase complex (*acsABCDE*) and formate dehydrogenase (*fdhAB*) for the WLP were absent in the complete genome of B11^T^, which makes syntrophic acetate oxidation highly improbable. Further experiments showed that B11^T^ was unable to grow with acetate, or glucose alone, but it could be maintained on a LS medium supplemented with 10 mM glucose and 0.5 g/L yeast extract (Figure S3a). Therefore, LS medium with 10 mM glucose and 0.5 g/L yeast extract was used as the basic culture medium to carry out physiological experiments. The experiments showed that isolate B11^T^ is anaerobic but was able to tolerate up to 2% oxygen (Figure S3b), and the genome analysis results indicated that it contains multiple genes for detoxification of reactive oxygen species (Table S6: peroxidase, alkyl hydroperoxide reductase, alkylhydroperoxidase, and superoxide reductase). It has an optimum growth temperature of 70 ºC (Figure S3c), optimal pH of 7.0 (Figure S3d), and optimal NaCl concentration of 10 g/L (Figure S3e). These traits are consistent with its environmental source in the deep subsurface within high-temperature brines associated with the Haoxian Oil Reservoir. Average nucleotide identity (ANI) analysis showed that the complete genome of isolate B11^T^ and MAG HX-AS.bin.3 from the acetate enrichment are the same species (98%) by using 95% ANI as the cut-off [47] (Figure S4). Therefore, the genome of strain B11^T^ and MAG HX-OS.bin.34, together with 46 high-quality *Atribacterota* genomes from other sources (Table S5), were subjected to further analyses to assess their diversity, physiology, and evolution, especially in acetate- and hydrocarbon-enriched environments.

### Phylogenomic analyses reveal two different *Atribacterota* in the Haoxian Oil Reservoir of Shengli Oilfield and support the division of *Atribacterota* into two major classes

Phylogenetic analysis confirmed that these 48 genomes separated into the two known major lineages of *Atribacterota* (Fig. 1e), the class *Atribacteria* (OP9) and the class informally named JS1 [2, 3]. Isolate B11^T^ branched from a basal node within the *Atribacteria* as a sister group to the family *Ca*. Caldatribacteriaceae. The phylogenetic position and distinctness of B11^T^ based on relative evolutionary divergence indicated that B11^T^ is a novel species of a new family within the order *Atribacterales* (Fig. 1e; Table S5). Here, we propose the name *Thermatribacter velox* B11^T^ as the first pure culture of the novel family *Thermatribacteraceae*. In contrast, MAG HX-OS.bin.34, derived from the natural oil sample, belongs to the class previously defined as JS1 [3]. We propose to name JS1 herein under the SeqCode as *Phoenicimicrobiia*, with the nomenclatural type being *Phoenicimicrobium oleiphilum* HX-OS.bin.34^TS^. AAI and ANI analyses were used in conjunction with the phylogenomic analysis to further understand the diversity of existing *Atribacterota* genomes (Supplementary information and Figure S5–S6). Our refined taxonomic classification resulted in 2 classes, 2 orders, 5 families, 13 genera, and 31 species represented by high-quality MAGs and complete genomes of isolates, which significantly expands the previously published three species in the *Atribacterota* (*Atribacter laminatus* RT761^T^, *Ca*. Caldatribacterium saccharofermentans, and *Ca*. Caldatribacterium californiense). Taxonomic names are proposed within protologues at the end of the main text and in Table S5.

### Genomic properties and functional annotation of *Atribacterota* genomes

**Fig. 2:**
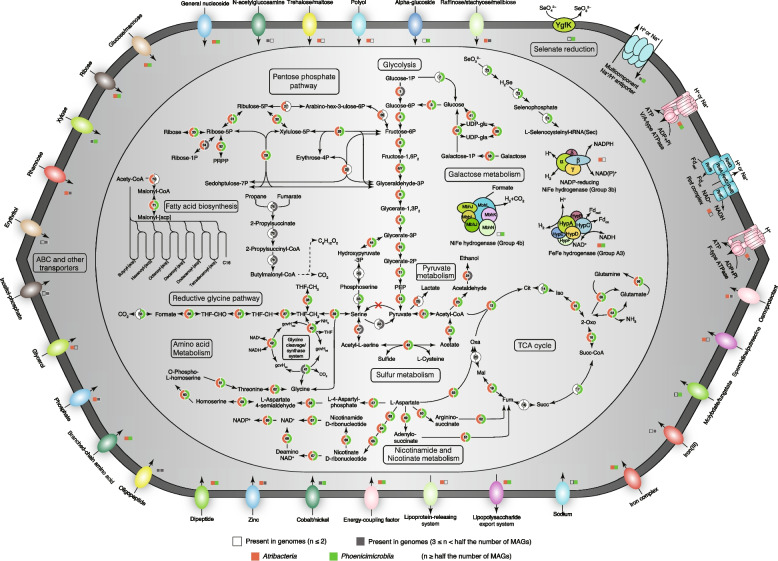
Overview of metabolic capabilities of the classes *Atribacteria* (OP9) and *Phoenicimicrobiia* (JS1). Different colors in gene circles represent genes present at the class level. PRPP: 5-Phospho-alpha-D-ribose 1-diphosphate, Cit: Citrate, Iso: Isocitrate, 2-Oxo: 2-Oxoglutarate, Succ-CoA: Succinyl-CoA, Succ: Succinate, Fum: Fumarate, Mal: Malate, Oxa: Oxaloacetate, THF: Tetrahydrofolate, THF-CHO: 10-Formyltetrahydrofolate, THF-CH: 5,10-Methenyltetrahydrofolate, THF-CH_2_: 5,10-Methylenetetrahydrofolate, THF-CH_3_: 5-Methyltetrahydrofolate, gcvH: glycine cleavage system H protein

*Atribacterota* genome sizes ranged from 1.2 Mb (Gw_SlDig_bin_137) to 3.1 Mb (*Atribacter laminatus* RT761^T^), with smaller genome sizes in the *Atribacteria* than in *Phoenicimicrobiia* (Wilcoxon, *p* < 0.05; Figure S7; Table S1). Principal coordinates analysis (PCoA) based on the KEGG orthologs (KOs) and Clusters of Orthologous Groups (COGs) showed that genomes clustered according to their taxonomy (Figure S8), underscoring the functional divergence of the two classes. Metabolic pathways inferred from the genomes revealed conserved central carbon metabolic pathways, including complete pathways for glycolysis, gluconeogenesis, pentose phosphate pathway, and the incomplete horseshoe-type tricarboxylic cycle, which is used to generate anabolic intermediates (Fig. 2; Table S6). Considering the absence of cytochrome c/bd oxidase and superoxide reductase in all genomes (Table S6), the *Atribacterota* are probably all strict anaerobes. COG categories that were significantly different between *Atribacteria* and *Phoenicimicrobiia* included those for motility (category N), carbohydrate transport and metabolism (category G), coenzyme transport and metabolism (category H), and signal transduction mechanisms (category T) (Figure S9). Genes for flagellum assembly and chemotaxis (category N) were found in all 15 genomes of the families *Thermatribacteraceae* (F1) and *Ca*. Caldatribacteriaceae (F2) within class *Atribacteria*, consistent with previous genomic predictions [6]; however, no flagellar components were detected in B11^T^ (Fig. 1b–d) and *Atribacter laminatus* RT761^T^ [2]. The enrichment of genes for carbohydrate transport and metabolism (category G) in *Atribacteria* is consistent with sugar catabolism by B11^T^ and *Atribacter laminatus* RT761^T^ and suggests the two classes of *Atribacterota* have different carbon metabolisms. Genes encoding bacterial microcompartments in *Atribacteria* (OP9) are significantly more widespread than in *Phoenicimicrobiia* (JS1). *Phoenicimicrobiia* only has two genomes (RBG_19FT_COMBO_35_14 and Bin1_14.8m) simultaneously containing COG4576 and COG4577 (Table S6). Furthermore, putative selenate reductase gene *ygfK* was only detected in *Phoenicimicrobiia*, indicating the potential serenate reduction coupled with respiration for energy metabolism. These results also imply functional differences between the two classes.

### Conserved carbohydrate fermentation in *Atribacteria* (OP9)

**Fig. 3:**
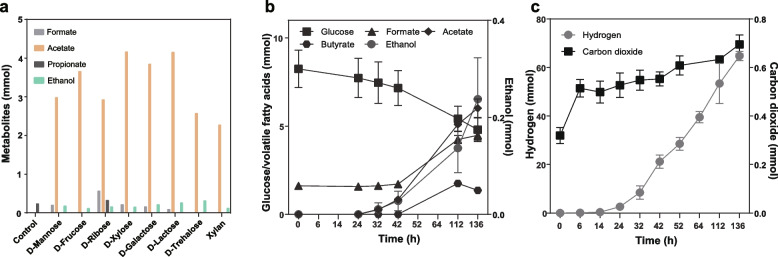
Carbohydrate fermentation and products of *Thermatribacter velox* B11^T^. **a** Volatile fatty acids and ethanol produced from saccharides and the polysaccharide xylan after 142 h incubation. Control, no extra substrate. **b** Fatty acids and ethanol production during fermentation of glucose. **c** Hydrogen and carbon dioxide production during fermentation of glucose

Analysis of the first high-quality *Atribacteria* genomes from lignocellulose enrichments suggested sugar fermentation, similar to the known physiology of *A. laminatus* RT761^T^, which ferments glucose, producing H_2_, acetate, CO_2_, and trace amounts of ethanol [3, 6]. The isolation of B11^T^ allowed additional experimentation on a member of a new family of *Atribacteria*. Considering the physiology of *A. laminatus* RT761^T^ and the presence of genes encoding NADP-reducing NiFe (Group 3b) hydrogenases, FeFe (Group A3 and C) hydrogenases (Figure S10–12), acetogenesis (*pta*, *ackA*, *acyP*, *eutE*, *adhP,* and *yiaY*), alcohol dehydrogenase (*adhP*, and *yiaY*), and Rnf complex (*rnfABCDEG*), we speculated that B11^T^ may ferment sugars to acetyl-CoA, which could then be further used for reoxidation of NADH via acetyl-CoA reduction to acetate, ethanol, and H_2_ (Fig. 2). To assess this prediction, we tested the growth of strain B11^T^ (70 ºC and pH 7) in media supplemented with different carbohydrates (d-mannose, d-fructose, d-ribose, d-xylose, d-galactose, d-lactose, d-trehalose, and xylan) adjusted to a final concentration of 10 mM (5 g/L for xylan) and checked for growth and the formation of formate, acetate, propionate, ethanol, H_2,_ and CO_2_. The results showed that d-mannose, d-fructose, d-ribose, d-xylose, d-galactose, and xylan can be utilized by B11^T^, similar to *Atribacter laminatus* RT761^T^ (Table S7), and fermentation products included acetate, H_2_, and CO_2_ (Fig. 3). These results support prior suggestions that sugar fermentation may be universal within the *Atribacteria* [2]. We also found that the number of glycoside hydrolase (GH) families was significantly higher in *Atribacteria* genomes than in *Phoenicimicrobiia* genomes (Figure S13). GHs may be used for depolymerization of xylan and other oligo- and polysaccharides. The near-universal presence of genes encoding GHs, NiFe Group 3b hydrogenases, FeFe Group A3 hydrogenases, acetogenesis, and Rnf complexes suggests sugar fermentation to acetate, H_2_, and CO_2_ is a common core metabolism in the class *Atribacteria* (Figure S14).

### Anaerobic hydrocarbon degradation might be prevalent in *Phoenicimicrobiia* (JS1)

**Fig. 4:**
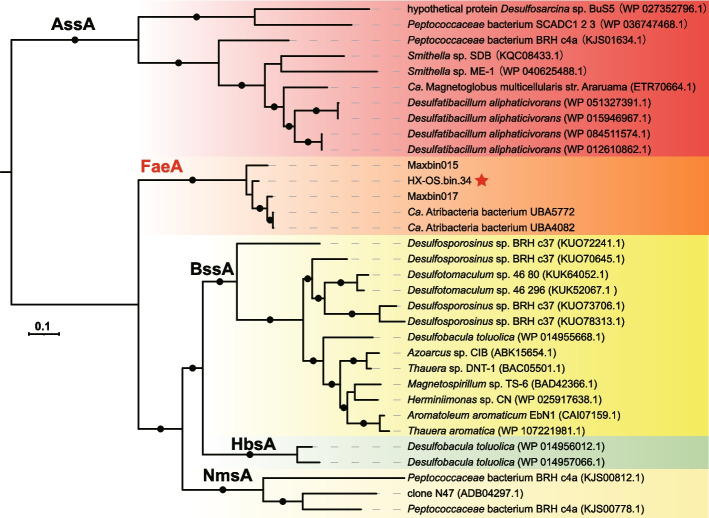
Phylogenetic tree of *Phoenicimicrobiia* putative FaeA. Reference sequences of AssA, BssA, HbsA, and HmsA were obtained from previous studies [1]. The genes were aligned using MUSCLE [39] with 100 iterations, and divergent regions were eliminated using TrimAL [40]. The IQ-Tree was used for phylogenetic inference [41], and the best model LG+F+I+G4 was well supported by Bayesian Information Criterion (BIC). Bootstrap values > 80 were shown in black dots

We next focused our attention on MAG HX-OS.bin.34, which belongs to the class *Phoenicimicrobiia* (JS1)*.* Putative fumarate-adding enzymes (FAE) were detected in the MAG HX-OS.bin.34, which is in line with a previous report suggesting the capacity of some members of *Phoenicimicrobiia* (JS1) to ferment short-chain *n-*alkanes [1]. However, this function in *Phoenicimicrobiia* has not been investigated experimentally. Fumarate addition to methyl groups via FAEs is a well-known mechanism to initiate anaerobic catabolism of hydrocarbons [55]. Genes encoding putative FAEs were also found in MAGs from wastewater and represented four non-monophyletic genus-level groups herein designated as *Oleincola* (G8), *Oleihabitans* (G9), *Phoenicimicrobium* (G10) and *Immundihabitans* (G11), all belonging to the class *Phoenicimicrobiia* (Fig. 1e, Table S5). The broad phylogenetic distribution of FAE-encoding MAGs and their presence in both oil reservoirs and wastewater suggests broad importance for hydrocarbon metabolism in *Phoenicimicrobiia* (Figure S15). Phylogenetic analyses showed that the putative alpha-subunits of FAE (*faeA*) from the *Phoenicimicrobiia* MAGs form a single clade that branched deeply among members of the fumarate reductase superfamily, which includes protein sequences encoded by *assA*, *bssA*, *nmsA*, and *hbsA* genes (Fig. 4). The monophyly of *faeA* homologs from *Phoenicimicrobiia* MAGs and synteny of the *faeABCDEF* gene cluster [1] suggests these genes were likely present in the common ancestor of the class *Phoenicimicrobiia* and extended by vertical inheritance. The presence of *faeABCDEF* explains why *Phoenicimicrobium oleiphilum* HX-OS.bin.34^TS^ was relatively abundant in the original sample from the Haoxian Oil Reservoir (Table S3).

### Reductive glycine pathway rather than the Wood-Ljungdahl pathway supports formate/CO_2_ assimilation and syntrophic acetate oxidation

The genetic capacity for syntrophic acetate oxidation or carbon fixation through the WLP was reported in *Atribacterota* genomes in previous studies [4, 9, 10]. Almost all *Atribacterota* genomes possess genes for formate-tetrahydrofolate ligase (*fhs*), methylenetetrahydrofolate dehydrogenase (*folD*), and methylenetetrahydrofolate reductase (*metF*), which together with *fdhAB*, constitutes the methyl-branch of the WLP. However, we note that only members of *Phoenicimicrobiia* (JS1) contain the *fdhAB* genes necessary for reducing CO_2_ to formate. Furthermore, only two *Atribacterota* genomes possess genes encoding the CODH/ACS complex (*acsA*, *acsB*, *acsC*, *acsD*, and *acsE*), the key genes for the carbonyl branch of WLP. These two genomes belong to a single genus-level group in the class *Atribacteria*, herein designated *Nitricultor* (G5) (Atribacterota bacterium CSSed162cmB 266 and Atribacterota bacterium CSSed11 91).

**Fig. 5:**
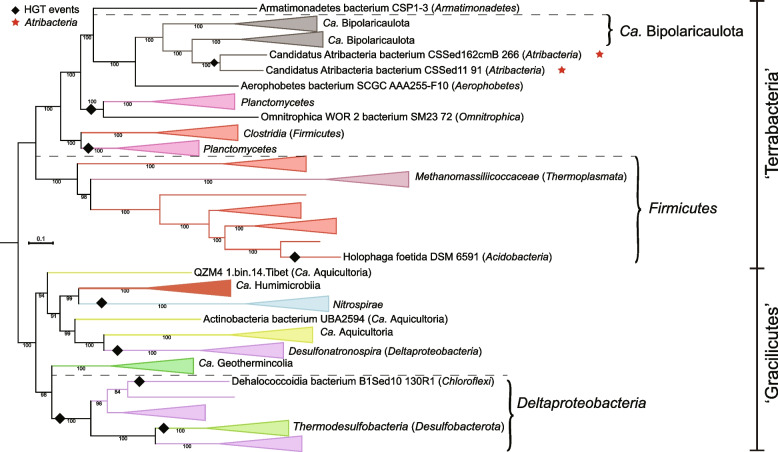
Phylogenetic tree based on concatenated AcsDABCE sequences. Maximum likelihood phylogeny of concatenated AcsDABCE sequences was performed as previous studies [29, 42]. The subunits of AcsD, AcsA, AcsB, AcsC, and AcsE were aligned using MUSCLE with 100 iterations [39], respectively. The alignments were concatenated by using a perl script (https://github.com/nylander/catfasta2phyml). IQ-Tree [41] was used for phylogenetic inference with parameters (-alrt 1000 -bb 1000 -nt AUTO). The best-fit model was LG+F+R8, which was well supported by the Akaike Information Criterion (AIC), Corrected Akaike Information Criterion (cAIC) and Bayesian Information Criterion (BIC). Phylogenetic tree was visualized and annotated using iTOL [66]

To further investigate the evolutionary history of the *Nitricultor* CODH/ACS complex, we reconstructed phylogenetic trees according to previous studies [29, 42]. The phylogenetic trees showed that the *Nitricultor* CODH/ACS complex proteins (*acsABCDE*) were nested within a set of homologs from the phylum ‘*Ca*. Bipolaricaulota’, suggesting these genes arose from a single horizontal gene transfer event (Fig. 5; Figure S16–S17). Given the absence of key genes encoding the methyl-branch (*fdhAB*) in *Atribacteria* and key genes encoding the carbonyl-branch (*acsABCDE*) in most *Atribacterota*, members of this phylum appear to be incapable of reducing CO_2_ or oxidizing acetate via the WLP. Considering experimental evidence that some *Atribacterota* became labeled after incubation of partially serpentinized rocks and sediments from the Mid-Atlantic Ridge with ^13^C-bicarbonate and H_2_ or with ^13^C-acetate [7], we explored the possibility that some *Phoenicimicrobiia* may have the genetic capacity to reduce CO_2_ to formate using *fdhAB*.

The only known natural carbon-fixation pathway that can assimilate formate is the reductive glycine pathway (RGP) [56]. Surprisingly, the key genes for the RGP (*fhs*, *folD*, *glyA*, *gcvT*, *gcvPA*, *gcvPB*, *pdhD*) as well as *fdhAB* were nearly universal in *Phoenicimicrobiia* and some *Atribacteria* genomes (which lack *fdhAB*), including isolate B11^T^, indicating that the RGP is likely the route for CO_2_/formate assimilation. In reverse, these enzymes could facilitate their participation in syntrophic acetate oxidation, rather than the previously reported WLP [4, 9, 10].

### Evolutionary history of syntrophic physiology in *Atribacterota*

**Fig. 6:**
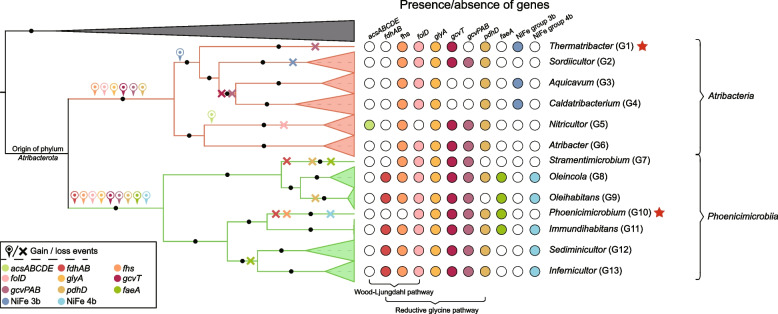
Evolutionary history of syntrophic function in *Atribacterota*. The Bayesian tree topology was determined by MrBayes [43] by using multiple sequence alignments of 16 ribosomal proteins. The results of the standard deviation of split frequencies (< 0.01), the potential scale reduction factor (PSRF = 1), and the effective sample size (ESS > 100) made the Bayesian tree highly reliable. The time-calibrating phylogenetic tree was further inferred by using R with the ape package. The evolutionary history of *Atribacterota* was inferred by using COUNT [50] as previously described [29, 51], and further confirmed by phylogenetic trees of each protein set

Following ancestral gene content reconstruction and phylogenetic analyses, we examined the gain and loss of genes relevant to syntrophic functions throughout the evolutionary history of *Atribacterota* (Fig. 6). As noted previously, genes for the CODH/ACS complex were largely absent in *Atribacterota* and were only gained from ‘*Ca*. Bipolaricaulota’ via HGT to the single genus *Nitricultor* (Fig. 5; Figure S16–S17). In contrast, the RGP and diverse hydrogenases are prevalent in extant *Atribacterota*, and the ancestral character state reconstruction indicated that these genes were ancestral within the phylum. Thus, we interpret that hydrogenases and the RGP are a part of the core metabolism of the *Atribacterota* and were critical for its evolution.

To gain more insight into the evolutionary history of these genes within the phylum, the protein sequences of the RGP enzymes and hydrogenases that supply reducing power for the RGP were selected for phylogenetic analyses: NiFe hydrogenase group 1–3 (Figure S10), NiFe hydrogenase group 4 (Figure S11), FeFe hydrogenase (Figure S12), *fdhA* (Fig. S18), *fhs* (Figure S19), *folD* (Figure S20), *glyA* (Figure S21), *gcvT* (Figure S22), *gcvPA* (Figure S23), *gcvPB* (Figure S24), and *pdhD* (Figure S25). If RGP-based metabolism was present in the last common ancestor of *Atribacterota*, it is expected that their RGP core proteins would be monophyletic. However, the phylogenetic trees revealed two distinct evolutionary trajectories between the classes *Atribacteria* and *Phoenicimicrobiia* (Figure S10–12; Figure S18–S25). Our results strongly suggest that the two classes of *Atribacterota* obtained genes for the RGP, hydrogen metabolism, hydrocarbon degradation, and sugar fermentation independently through lateral gene transfer after their divergence (Fig. 6). Given this set of common core metabolic genes, but with different evolutionary histories, we developed genome-resolved models based on MAGs derived from microbial communities in the hydrocarbon-rich oil reservoir sample (HS-OX) and the acetate enrichment (HS-AX) to further understand potential ecological roles of *Phoenicimicrobium* and B11^T^.

### The ecological function of *Phoenicimicrobium oleiphilum* (JS1) in hydrocarbon-enriched environments

**Fig. 7:**
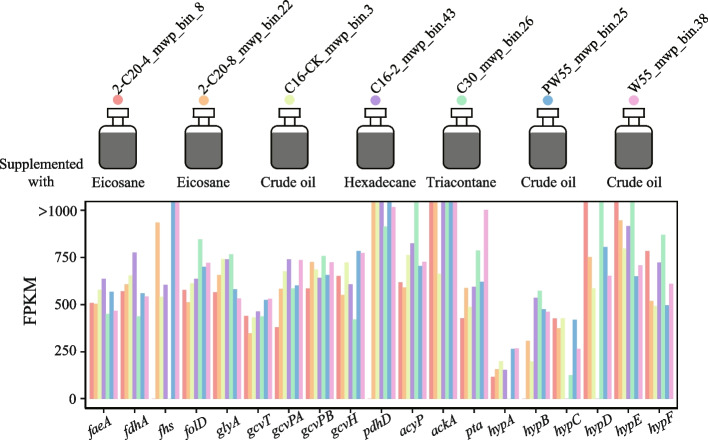
Laboratory enrichments and metatranscriptomic analysis of *Phoenicimicrobium oleiphilum* from crude oil-, hexadecane-, eicosane-, and triacontane-enriched samples. The laboratory enrichments were performed in anoxic PRM at 55 °C with oily sludge as inoculum. The supplementary substrates were added by 1 ml/L hexadecane, 2 ml/L eicosane, 0.6 g/L triacontane, and 200 g/L oily sludge, respectively

In the original sample from the Haoxian Oil Reservoir, the dominant member of the *Atribacterota* was *Phoenicimicrobium oleiphilum* HX-OS.bin.34^TS^, with a relative metagenomic read abundance of 2.33% (Table S3). Metatranscriptomic reads were obtained directly from samples from the Haoxian Oil Reservoir mapping to the HX-OS.bin.34 genome were also relatively high at 1.32% (Table S8), suggesting it is highly active. Almost all the genes encoded by HX-OS.bin.34 were highly expressed (Table S8), including the genes for FAE, RGP, acetogenesis, and hydrogenases, which is consistent with the inferred metabolism of alkane degradation. The role of *Phoenicimicrobium oleiphilum* in *n*-alkanes degradation was further confirmed by establishing short-term anaerobic enrichment cultures with samples from the Shengli Oilfield that were amended with crude oil, hexadecane, eicosane, or triacontane (Fig. 7; Table S9–10). MAGs with > 98% ANI values to *Phoenicimicrobium oleiphilum* HX-OS.bin.34^TS^ and associated metatranscriptome reads were obtained from each enrichment culture. To avoid biases in estimating gene expression, we selected seven high-quality MAGs representing the dominant taxa from each enrichment culture as reference genomes. High numbers of transcript read obtained from all of these enrichment cultures mapped to the key genes in *Phoenicimicrobium oleiphilum* for *n*-alkanes degradation, RGP, acetogenesis, and hydrogenases, suggesting an active role in the degradation of a variety of *n*-alkanes and producing H_2_, CO_2_, and acetate (Figure S15). These results extend the role of JS1 to the fermentation of larger *n*-alkanes rather than only short-chain *n*-alkanes, which was suggested previously [1]. The expression of these genes in samples from the Haoxian Oil Reservoir and these enrichment cultures imply a role for *Phoenicimicrobium oleiphilum* in the fermentation of hexadecane, eicosane, and triacontane to H_2_, CO_2,_ and acetate.

**Fig. 8:**
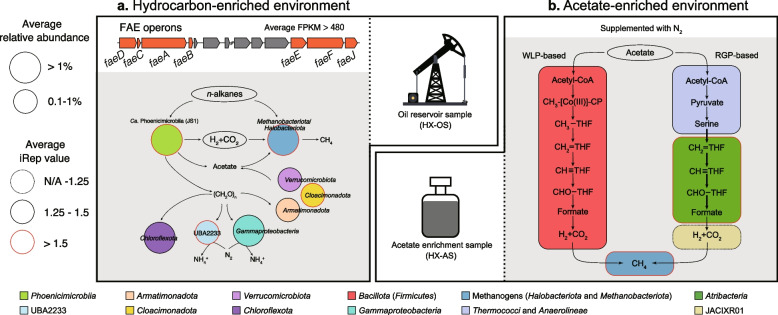
Metabolic models in the hydrocarbon- and acetate-enriched environments. The metabolic models are based on the 33 representative genomes. **a** Genome and metatranscriptome resolved model in the hydrocarbon-enriched environments. **b** Metagenome and genome resolved model in the acetate-enriched environments

Based on the annotation of 21 representative MAGs (Table S3) from the HX-OS sample, the flux of carbon and electrons from *n*-alkanes to H_2_, CO_2_, acetate, and CH_4_ may require primary hydrocarbon degraders such as *Phoenicimicrobium oleiphilum* HX-OS.bin.34^TS^ and *Methanoliparum* [8] as well as secondary metabolisms to obtain redox balance, including acetoclastic methanogens such as *Methanothrix* [57], hydrogenotrophic methanogens such as *Methanothermus* and *Methanothermobacter* [58, 59], and hydrogenotrophic and methylotrophic methanogens *Methanobacteriaceae* [58] to form a syntrophic network (Fig. 8a).

### Metabolic handoffs in a thermophilic acetate-oxidizing enrichment

To better understand the ecology of *Thermatribacter velox* B11^T^ in the acetate-amended enrichment culture from which it was isolated, we examined the metagenome of the culture and considered syntrophy and metabolic handoffs [60–63], noting that very little is known about anaerobic processing of acetate at high temperatures (75 ºC). Only a single MAG encoded the complete gene set for methyl coenzyme M reductase, *mcrABCDG*, the hydrogenotrophic methanogen *Methanothermobacter* [58] (Table S3), whose relative abundance increased from 6.53 (HX-OS) to 15.49% (HX-AS) after acetate enrichment. The complete gene sets for both WLP and RGP were detected in the metagenome, suggesting that two separate syntrophic acetate degradation pathways (SADPs) might operate, with a single methanogen as a hydrogen sink (Fig. 8b) [64]. The relative abundance of *Bacillota* (*Firmicutes*) increased from 0.01 (HX-OS) to 6.55% (HX-AS) after acetate enrichment. These *Bacillota* included the genus *Thermoanaerobacter* and uncultivated members of the *Moorellales*, and most encoded the potential for complete oxidation of acetate to H_2_ and CO_2_ via the WLP [64]. Although more than a quarter of the *Bacillota* cells were replicating based on bias in the number of reads near the origin of replication (iRep > 1.25; Table S3) [34], the fastest replicating organism was *Thermatribacter velox* B11^T^ (iRep > 2). Considering B11^T^ was unable to grow as a pure culture with acetate alone and the presence of the RGP and hydrogenases, we speculate that it may participate in the partially syntrophic oxidation of acetate. Given its fast replication rate, we propose that the RGP-based SADP may compete with the WLP-based SADP under these conditions. Other organisms in the acetate enrichment may provide common goods for acetate oxidation or may consume metabolic intermediates or necromass. For example, only a single MAG assigned to the genus *Thermococcus*_A encodes the key genes necessary for serine biosynthesis, using a single enzyme with both D-serine dehydratase and racemase domains (Figure S26). Several other MAGs encoded genes for formate dehydrogenase (Table S3), suggesting formate may be an important metabolic handoff, which is in line with similar findings from other hydrothermal environments [63].

## Conclusions

*Atribacterota* is a globally distributed phylum but previously only one pure culture, *Atribacter laminatus* RT761^T^, and two described species from genomic assemblies *Ca*. Caldatribacterium saccharofermentans and *Ca*. Caldatribacterium californiense have been described [2, 6]. Here, we isolated a second member of the phylum from a terrestrial oil reservoir, *Thermatribacter velox* B11^T^, which is the first pure culture in the newly described family *Thermatribacteraceae.* This species was capable of fermenting a variety of carbohydrates, particularly d-mannose, d-fructose, d-ribose, d-xylose, d-galactose, d-trehalose, and xylan, with subsequent formation of acetate, ethanol, H_2,_ and CO_2_ as end products, broadening the scope of carbohydrate fermentation within the class *Atribacteria*. Yet, given its enrichment in acetate-fed cultures, and gene presence of hydrogenases and partially RGP in B11^T^, we speculate that B11^T^ and likely many other *Atribacteria* may participate in the partially RGP-based SADP. This metabolic flexibility may allow members of the *Atribacteria* to alternatively ferment either primary biomass substrates (i.e., poly-, oligo-, and monosaccharides) when available or simple substrates produced geochemically or through fermentation, which may be more reliable in the anoxic, carbon-rich environments where *Atribacteria* thrive.

A different species of *Atribacterota*, herein named *Phoenicimicrobium oleiphilum* HX-OS.bin.34^TS^, was more abundant than *Thermatribacter velox* B11^T^ in the natural oil reservoir samples. We noted that *Phoenicimicrobium oleiphilum* and several other genomes from class JS1, herein named *Phoenicimicrobiia*, encode fumarate-adding enzymes. These genes, together with *mcmLS*, NiFe hydrogenase Group 4b, and FeFe hydrogenase Group A3 strongly suggest a role in alkane degradation. In support of this, we were able to cultivate *Phoenicimicrobium oleiphilum* with crude oil or a variety of *n*-alkanes and demonstrate the presence of transcripts in these cultures mapping to key metabolic genes, including *faeABCDEF,* RGP, and hydrogenases. Phylogenetic analyses and synteny of *faeABCDEF* subunits suggested that anaerobic hydrocarbon degradation was ancestral and may be widespread in *Phoenicimicrobiia*. Furthermore, we also note the absence of the WLP in *Phoenicimicrobiia*, but note the universal presence of the RGP as a metabolic hub for both acetate oxidation and CO_2_/formate assimilation. The phylogenies of hydrogenases, CODH/ACS complex, RGP, and FaeA are overall congruent with the phylogenetic divergence of *Atribacteria* and *Phoenicimicrobiia*, indicating an apparent lateral gene transfer after their divergence and vertical inheritance in *Atribacteria* and *Phoenicimicrobiia* respectively. The reconstruction of the metabolic networks revealed the likely roles of distinct members of the *Atribacterota* in hydrocarbon- and acetate-rich environments within syntrophic metabolic networks. We believe that these novel insights into the diversity, physiology, ecology, and evolution of *Atribacterota* will contribute to further laboratory cultivation and promote more incisive studies of their syntrophic biology in other habitats worldwide.

## Protologues

### Description of *Thermatribacter* gen. nov. (G1, ICNP)

*Thermatribacter* (Therm.a.tri.bac´ter. Gr. masc. adj. *thermos*, hot; N.L. masc. n. *Atribacter*, a bacterial genus; N.L. masc. n. *Thermatribacter*, a thermophilic *Atribacter*).

Anaerobic, extremely thermophilic, Gram-negative, non-motile, and non-spore-forming fusiform rod or ovoid shaped cells. Major cellular fatty acids are C_16:0_, C_18:0_ and *iso*-C_15:0_. Acetate, ethanol, hydrogen and carbon dioxide are produced from glucose.

Type species: *Thermatribacter velox*.

### Description of *Thermatribacter velox* sp. nov. (S1, ICNP)

*Thermatribacter velox* (ve´lox. L. masc. adj. *velox*, rapid).

Shows the following characteristics in addition to those given for the genus: colonies are white, opaque, circular, and flat colony with entire margin and 2–5 mm in diameter. Cells have a size of 0.4–0.5 μm in width and 0.6–1.9 μm in length. No flagellum is observed. Grows at 45–75 ºC, at pH 6.0–7.6 and in the presence of 0–40 g/L NaCl, with the optimal growth at 70 ºC, pH 6.5–7.0 with 10 g/L NaCl. Yeast extract is required for growth. Growth occurs with hexose (ribose, xylose), pentose (glucose, galactose, fructose, and trehalose), alditol (mannitol), disaccharide (lactose), and polysaccharide (xylan), and weakly used formate and fumarate.

The type strain B11^T^ (=CCAM 969^T^=JCM 39351^T^) was isolated from an oil sludge collected from an oil tank of Shengli Oilfield in China.

### Description of *Thermatribacteraceae* fam. nov. (F1, ICNP)

*Thermatribacteraceae* (Therm.a.tri.bac.te.ra.ce´ae. N.L. masc. n. *Thermatribacter*, a bacterial genus; -*aceae*, ending to denote a family; N.L. pl. fem. n. *Thermatribacteraceae*, the *Thermatribacter* family).

The description is the same as that of the genus *Thermatribacter*.

Type genus: *Thermatribacter*.

### Description of *Phoenicimicrobium* gen. nov. (G10, SeqCode)

*Phoenicimicrobium* (Phoe.ni.ci.mi.cro’bi.um. L. masc. n. *phoenix*, Phoenix, N.L. neut. n. *microbium*, a microbe; N.L. neut. n. *Phoenicimicrobium*, a microbe of Phoenix (a mythological symbol of ancient Egypt), referring to as an immortal or resurrected microbe).

Type species: *Pheonicimicrobium oleiphilum*.

### Description of *Phoenicimicrobium oleiphilum* sp. nov. (S23, SeqCode)

*Phoenicimicrobium oleiphilum* (o.le.i´phi.lum. L. neut. n. *oleum*, oil; N.L. neut. adj. suff. *-philum*, loving; N.L. neut. n. *oleiphilum*, oil-loving, referring to the source of the genome from an oil reservoir).

Type genome: HX_OS.bin.34 (PRJNA970932), obtained from the metagenome assembly of an oil reservoir sample.

### Description of *Phoenicimicrobiaceae* fam. nov. (F5, SeqCode)

*Phoenicimicrobiaceae* (Phoe.ni.ci.mic.ro.bi.a.ce’ae. N.L. neut. n. *Phoenicimicrobium*, type genus of the family; L. suff. –*aceae*, ending to denote a family; N.L. fem. pl. n. *Phoenicimicrobiaceae*, the *Phoenicimicrobium* family).

The family at present contains four genera *Phoenicimicrobium* gen. nov., *Immunidihabitans* gen. nov., *Sediminicultor* gen. nov. and *Infernicultor* gen. nov.

Type genus: *Phoenicimicrobium*.

### Description of *Pheonicimicrobiales* ord. nov. (O2, SeqCode)

*Phoenicimicrobiales* (Phoe.ni.ci.mi.cro.bi.a’les. N.L. neut. n. *Phoenicimicrobium*, type genus of the order; L. suff. –*ales*, ending to denote an order; N.L. fem. pl. n. *Phoenicimicrobiales*, the *Phoenicimicrobium* order).

The order *Phoenicimicrobiales* comprised of two families *Pheonecimicrobiaceae* fam. nov. and *Stramentimicrobiaceae* fam. nov.

Type genus: *Phoenicimicrobium*.

### Description of *Phoenicimicrobiia* class. nov. (C2, SeqCode)

*Phoenicimicrobiia* (Phoe.ni.ci.mic.ro.bi’i.a. N.L. fem. pl. n. *Phoenicimicrobium* type genus of the class; L. suff. –*ia*, ending to denote a class; N.L. neut. pl. n. *PhoeniciSmicrobiia*, the class of the genus *Phoenicimicrobium*).

The description of the class is the same as for the order *Phoenicimicrobiales*.

Type genus: *Phoenicimicrobium*.

### Supplementary Information

Additional file 1: Figure S1. Diagram of enrichment and isolation of *Atribacterota*. Figure S2. Acetate degradation and methane production in the extremophilic enrichment HX-AS during semi-continuous incubation. Figure S3. Physiological experiments of *Thermatribacter* *velox* B11^T^. Figure S4. Genomic similarity of B11 and HX-AS.bin.3. Figure S5. Average amino acid identity (AAI) shared among *Atribacterota* genomes. Figure S6. Average nucleotide identity (ANI) shared among *Atribacterota* genomes. Figure S7. Difference in assembled genome size of *Atribacteria* and *Phoenicimicrobiia*. Figure S8. Principal coordinates analyses (PCoA) based on Clusters of Orthologous Groups (COGs) and KEGG Orthologs (KOs). Figure S9. Clusters of Orthologous Groups (COG) categories of *Atribacteria* and *Phoenicimicrobiia*. Figure S10. Phylogenetic tree of groups 1, 2 and 3 [NiFe] hydrogenases catalytic subunits. Figure S11. Phylogenetic tree of [NiFe] hydrogenase group 4 and related complexes. Figure S12. Phylogenetic tree of [FeFe] hydrogenase. Figure S13. Different Carbohydrate-active enzyme families (CAZy) and their distribution in *Atribacteria* and *Phoenicimicrobiia*. Figure S14. Schematic view of genes involved in sugar fermentation of *Atribacteria*. Figure S15. Schematic view of genes involved in hydrocarbon metabolism of *Phoenicimicrobiia*. Figure S16. Phylogenetic tree based on concatenated *acsAB* protein sequences. Figure S17. Phylogenetic tree based on concatenated *acsABC* protein sequences. Figure S18. Phylogenetic tree of *fdhA* protein sequences. Figure S19. Phylogenetic tree of *fhs* protein sequences. Figure S20. Phylogenetic tree of *folD* protein sequences. Figure S21. Phylogenetic tree of *glyA* protein sequences. Figure S22. Phylogenetic tree of *gcvT* protein sequences. Figure S23. Phylogenetic tree of *gcvPA* protein sequences. Figure S24. Phylogenetic tree of *gcvPB* protein sequences. Figure S25. Phylogenetic tree of *pdhD* protein sequences. Figure S26. D-serine dehydratase domain and racemase domain from the class *Thermococci*. Supplementary Text.

Additional file 2: Table S1. Genomic information of *Atribacterota*. Table S2. Relative abundances of phyla in HaoXian oil reservoir and acetate enrichment samples. Table S3. The relative abundance, iRep value and functional annotation of 33 representative species. Table S4. Cellular fatty acid composition of *Thermatribacter* *velox* B11^T^ and the unique type strain *Atribacter* *laminatus* RT761^T^ in the phylum *Atribacterota*. Table S5. Brief nomenclature of *Atribacterota* species. Table S6. List of genes and features presented in Fig. 2 of the main text. Table S7. Differential traits of *T. velox* B11^T^ and type strain *A*. *laminatus* RT761^T^ representing the family *Atribacteraceae* of phylum *Atribacterota*. Table S8. Metatranscriptomic analysis of HX-OS, only the genes from the MAG HX-OS.bin.34 were listed. Table S9. Metagenomic and metatranscriptomic sequencing from crude oil-, hexadecane-, eicosane- and triacontane-enriched samples. Table S10. Metatranscriptomic analysis of seven MAGs from crude oil-, hexadecane-, eicosane- and triacontane-enriched samples that are listed in Table S9.

## Data Availability

The MAGs described in this paper have been deposited under NCBI PRJNA970932. The complete genome sequence of *Thermatribacter velox* B11^T^ has been deposited under NCBI CP121689.

## Abbreviations

CODH/ACS: Carbon monoxide dehydrogenase/acetyl-CoA synthase complex
WLP: Wood-Ljungdahl pathway
RGP: Reductive glycine pathway
SAGs: Single-amplified genomes
MAGs: Metagenome-assembled genomes
ANI: Average nucleotide identity
AAI: Average amino acid identity
GTDB: Genome Taxonomy Database
iRep: Index of replication
