# Supplementary material for: Cultivation of novel Atribacterota from oil well provides new insight into their diversity, ecology, and evolution in anoxic, carbon-rich environments
Source: Microbiome. 2024 Jul 6;12:123. doi: 10.1186/s40168-024-01836-7 (PMC11227167; doi:10.1186/s40168-024-01836-7)
Supplement: Supplementary file 2 — Additional file 1: Figure S1. Diagram of enrichment and isolation of Atribacterota. Figure S2. Acetate degradation and methane production in the extremophilic enrichment HX-AS during semi-continuous incubation. Figure S3. Physiological experiments of Thermatribacter velox B11T. Figure S4. Genomic similarity of B11 and HX-AS.bin.3. Figure S5. Average amino acid identity (AAI) shared among Atribacterota genomes. Figure S6. Average nucleotide identity (ANI) shared among Atribacterota genomes. Figure S7. Difference in assembled genome size of Atribacteria and Phoenicimicrobiia. Figure S8. Principal coordinates analyses (PCoA) based on Clusters of Orthologous Groups (COGs) and KEGG Orthologs (KOs). Figure S9. Clusters of Orthologous Groups (COG) categories of Atribacteria and Phoenicimicrobiia. Figure S10. Phylogenetic tree of groups 1, 2 and 3 [NiFe] hydrogenases catalytic subunits. Figure S11. Phylogenetic tree of [NiFe] hydrogenase group 4 and related complexes. Figure S12. Phylogenetic tree of [FeFe] hydrogenase. Figure S13. Different Carbohydrate-active enzyme families (CAZy) and their distribution in Atribacteria and Phoenicimicrobiia. Figure S14. Schematic view of genes involved in sugar fermentation of Atribacteria. Figure S15. Schematic view of genes involved in hydrocarbon metabolism of Phoenicimicrobiia. Figure S16. Phylogenetic tree based on concatenated acsAB protein sequences. Figure S17. Phylogenetic tree based on concatenated acsABC protein sequences. Figure S18. Phylogenetic tree of fdhA protein sequences. Figure S19. Phylogenetic tree of fhs protein sequences. Figure S20. Phylogenetic tree of folD protein sequences. Figure S21. Phylogenetic tree of glyA protein sequences. Figure S22. Phylogenetic tree of gcvT protein sequences. Figure S23. Phylogenetic tree of gcvPA protein sequences. Figure S24. Phylogenetic tree of gcvPB protein sequences. Figure S25. Phylogenetic tree of pdhD protein sequences. Figure S26. D-serine dehydratase domain and racemase dom [file 40168_2024_1836_MOESM1_ESM.docx]

**Supplementary Information**

**Cultivation of novel *Atribacterota* from oil well provides new insight into their diversity, ecology, and evolution in anoxic, carbon-rich environments**

**Jian-Yu Jiao^1†^, Shi-Chun Ma^2†^, Nimaichand Salam^1,3^, Zhuo Zhou^2^, Zheng-Han Lian^1^, Li Fu^2^, Ying Chen^1^, Cheng-Hui Peng^2^, Yu-Ting OuYang^1^, Hui Fan^2^, Ling Li^2^, Yue Yi^2^, Jing-Yi Zhang^1^, Jing-Yuan Wang^2^, Lan Liu^1^, Lei Gao^10^, Aharon Oren^4^, Tanja Woyke^5,6^, Jeremy A. Dodsworth^7^, Brian P. Hedlund^8,9^*, Wen-Jun Li^1,10^* and Lei Cheng^2^***

^1^State Key Laboratory of Biocontrol, Guangdong Provincial Key Laboratory of Plant Resources and Southern Marine Science and Engineering Guangdong Laboratory (Zhuhai), School of Life Sciences, Sun Yat-Sen University, Guangzhou, 510275, PR China

^2^Key Laboratory of Development and Application of Rural Renewable Energy, Biogas Institute of Ministry of Agriculture and Rural Affairs, Chengdu, 610000, PR China

^3^Bioinformatics Laboratory, Transdisciplinary Biology, Rajiv Gandhi Centre for Biotechnology (RGCB), Thiruvananthapuram, 695011, India

^4^Department of Plant and Environmental Sciences, The Alexander Silberman Institute of Life Sciences, The Edmond J. Safra Campus, The Hebrew University of Jerusalem, Jerusalem, 9190401, Israel

^5^DOE Joint Genome Institute, Lawrence Berkeley National Laboratory, Berkeley, California, USA

^6^University of California Merced, Life and Environmental Sciences, Merced, California, USA

^7^California State University, San Bernardino, California, USA

^8^School of Life Sciences, University of Nevada Las Vegas, Las Vegas, NV 89154, USA

^9^Nevada Institute of Personalized Medicine, University of Nevada Las Vegas, Las Vegas, NV 89154, USA

^10^State Key Laboratory of Desert and Oasis Ecology, Key Laboratory of Ecological Safety and Sustainable Development in Arid Lands, Xinjiang Institute of Ecology and Geography, Chinese Academy of Sciences, Urumqi, 830011, PR China

^†^These authors contributed equally to this work.

**Correspondence:**

Lei Cheng: chenglei@caas.cn; Wen-Jun Li: liwenjun3@mail.sysu.edu.cn; Brian P. Hedlund, E-mail: brian.hedlund@unlv.edu

Table of Contents

[Supplementary Figures 5](#_Toc151757337)

[**Fig. S1. Diagram of enrichment and isolation of *Atribacterota*.** 5](#_Toc151757338)

[**Fig. S2. Acetate degradation and methane production in the extremophilic enrichment HX-AS during semi-continuous incubation.** 6](#_Toc151757339)

[**Fig. S3. Physiological experiments of *Thermatribacter velox* B11^T^.** 7](#_Toc151757340)

[**Fig. S4. Genomic similarity of B11 and HX-AS.bin.3.** 8](#_Toc151757341)

[**Fig. S5. Average amino acid identity (AAI) shared among *Atribacterota* genomes.** 9](#_Toc151757342)

[**Fig. S6. Average nucleotide identity (ANI) shared among *Atribacterota* genomes.** 10](#_Toc151757343)

[**Fig. S7. Difference in assembled genome size of *Atribacteria* and *Phoenicimicrobia*.** 11](#_Toc151757344)

[**Fig. S8. Principal coordinates analyses (PCoA) based on Clusters of Orthologous Groups (COGs) and KEGG Orthologs (KOs).** 12](#_Toc151757345)

[**Fig. S9. Clusters of Orthologous Groups (COG) categories of *Atribacteria* and *Phoenicimicrobia*.** 13](#_Toc151757346)

[**Fig. S10. Phylogenetic tree of groups 1, 2 and 3 [NiFe] hydrogenases catalytic subunits.** 14](#_Toc151757347)

[**Fig. S11. Phylogenetic tree of [NiFe] hydrogenase group 4 and related complexes.** 15](#_Toc151757348)

[**Fig. S12. Phylogenetic tree of [FeFe] hydrogenase.** 16](#_Toc151757349)

[**Fig. S13. Different CAZy families and their distribution in *Atribacteria* and *Phoenicimicrobia*.** 17](#_Toc151757350)

[**Fig. S14. Schematic view of genes involved in sugar fermentation of *Atribacteria*.** 18](#_Toc151757351)

[**Fig. S15. Schematic view of genes involved in hydrocarbon metabolism of *Phoenicimicrobiia*.** 19](#_Toc151757352)

[**Fig. S16. Phylogenetic tree based on concatenated AcsAB sequences.** 20](#_Toc151757353)

[**Fig. S17. Phylogenetic tree based on concatenated AcsABC sequences.** 21](#_Toc151757354)

[**Fig. S18. Phylogenetic tree of FdhA protein sequences.** 22](#_Toc151757355)

[**Fig. S19. Phylogenetic tree of Fhs protein sequences.** 23](#_Toc151757356)

[**Fig. S20. Phylogenetic tree of FolD protein sequences.** 24](#_Toc151757357)

[**Fig. S21. Phylogenetic tree of GlyA protein sequences.** 25](#_Toc151757358)

[**Fig. S22. Phylogenetic tree of GcvT protein sequences.** 26](#_Toc151757359)

[**Fig. S23. Phylogenetic tree of GcvPA protein sequences.** 27](#_Toc151757360)

[**Fig. S24. Phylogenetic tree of GcvPB protein sequences.** 28](#_Toc151757361)

[**Fig. S25. Phylogenetic tree of PdhD protein sequences.** 29](#_Toc151757362)

[**Fig. S26. D-serine dehydratase domain and racemase domain from the classes *Thermococci*.** 30](#_Toc151757363)

[Supplementary Text 31](#_Toc151757364)

[1. Physiology of *Thermatribacter velox* B11^T^. 31](#_Toc151757365)

[2. Genomic-based taxonomy of *Atribacterota*. 31](#_Toc151757366)

[3. Nomenclature of members of *Atribacterota*. 31](#_Toc151757367)

# Supplementary Figures

**
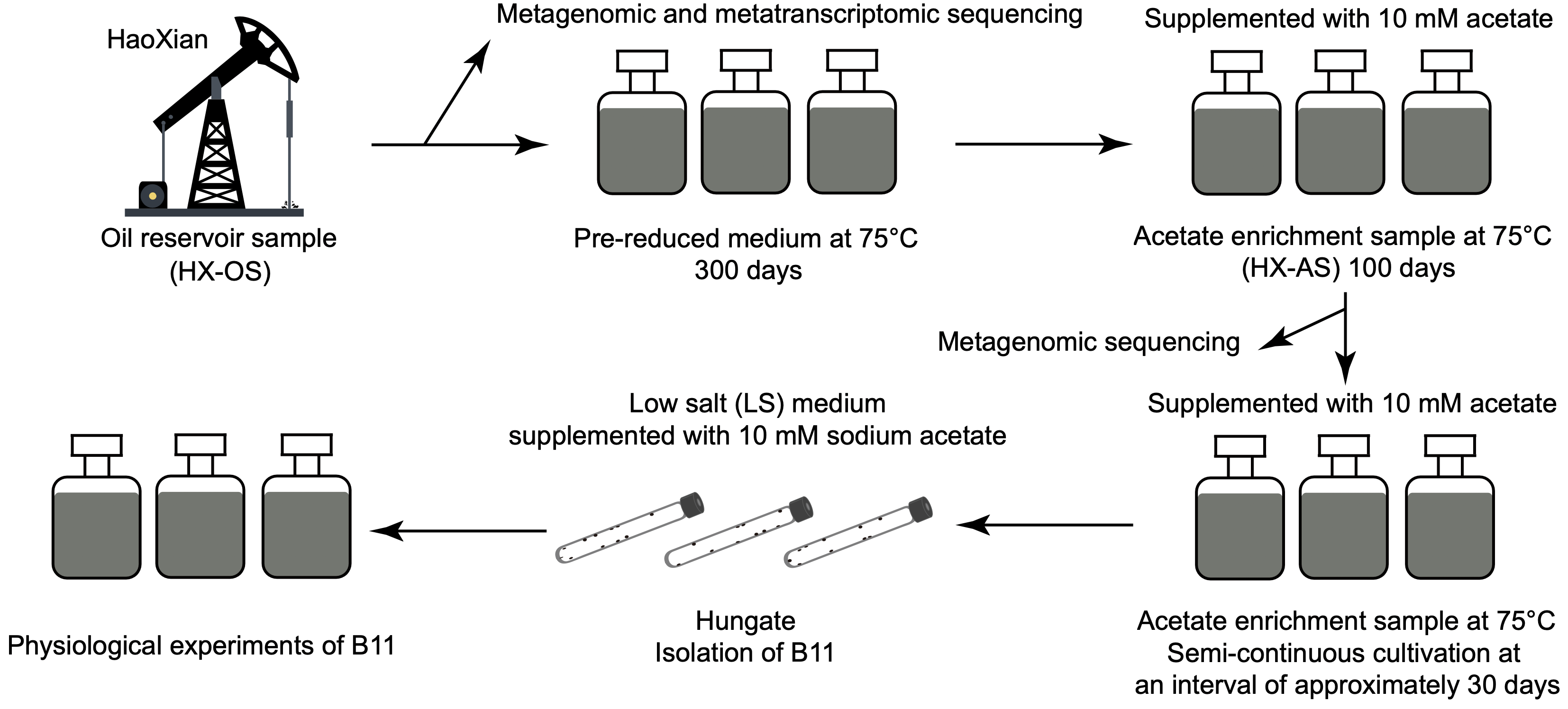
**

**Fig. S1. Diagram of enrichment and isolation of *Atribacterota*.**

**
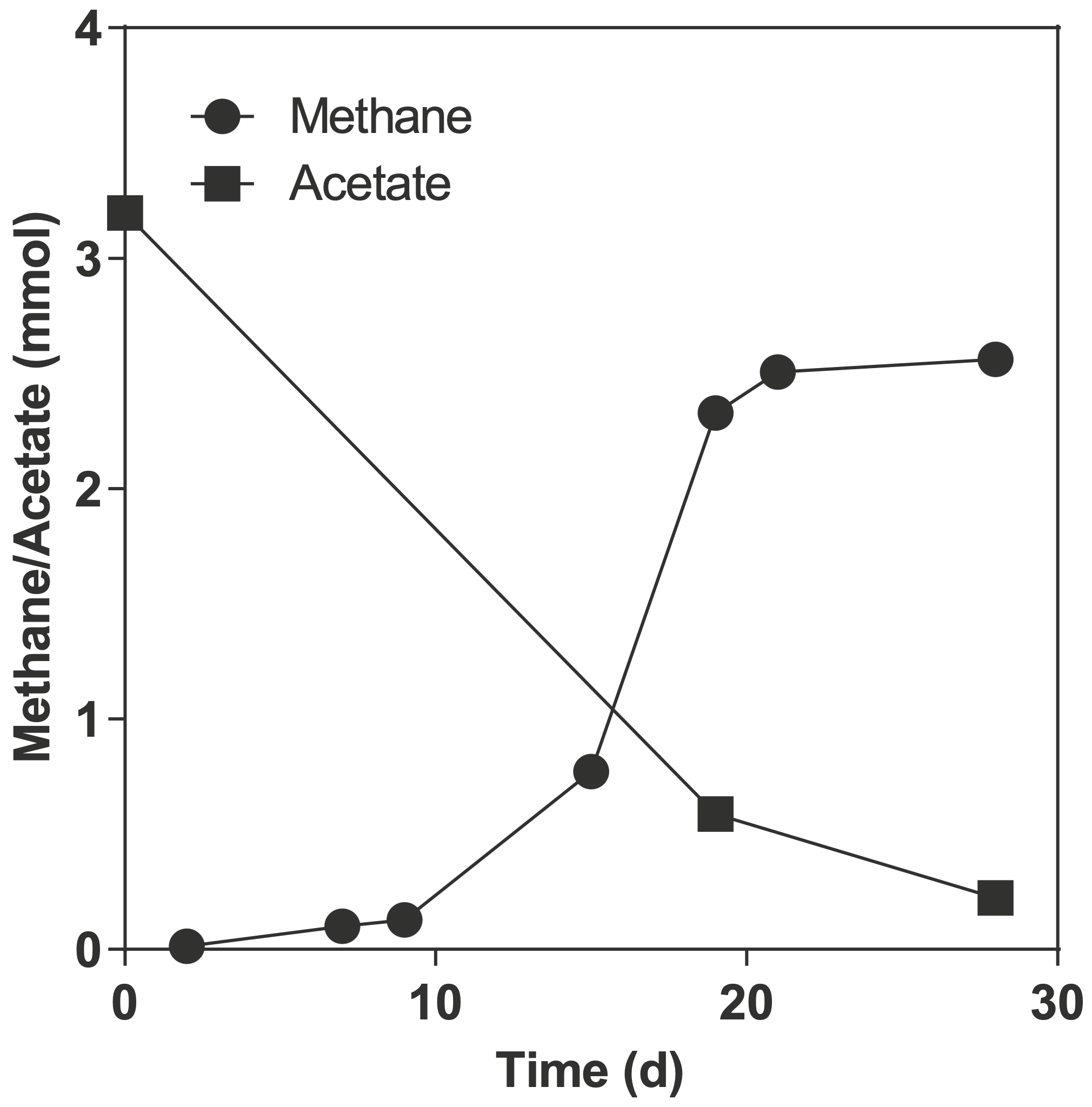
**

**Fig. S2. Acetate degradation and methane production in the extremophilic enrichment HX-AS during semi-continuous incubation.** The concentrations of CH_4_ and acetate were measured.


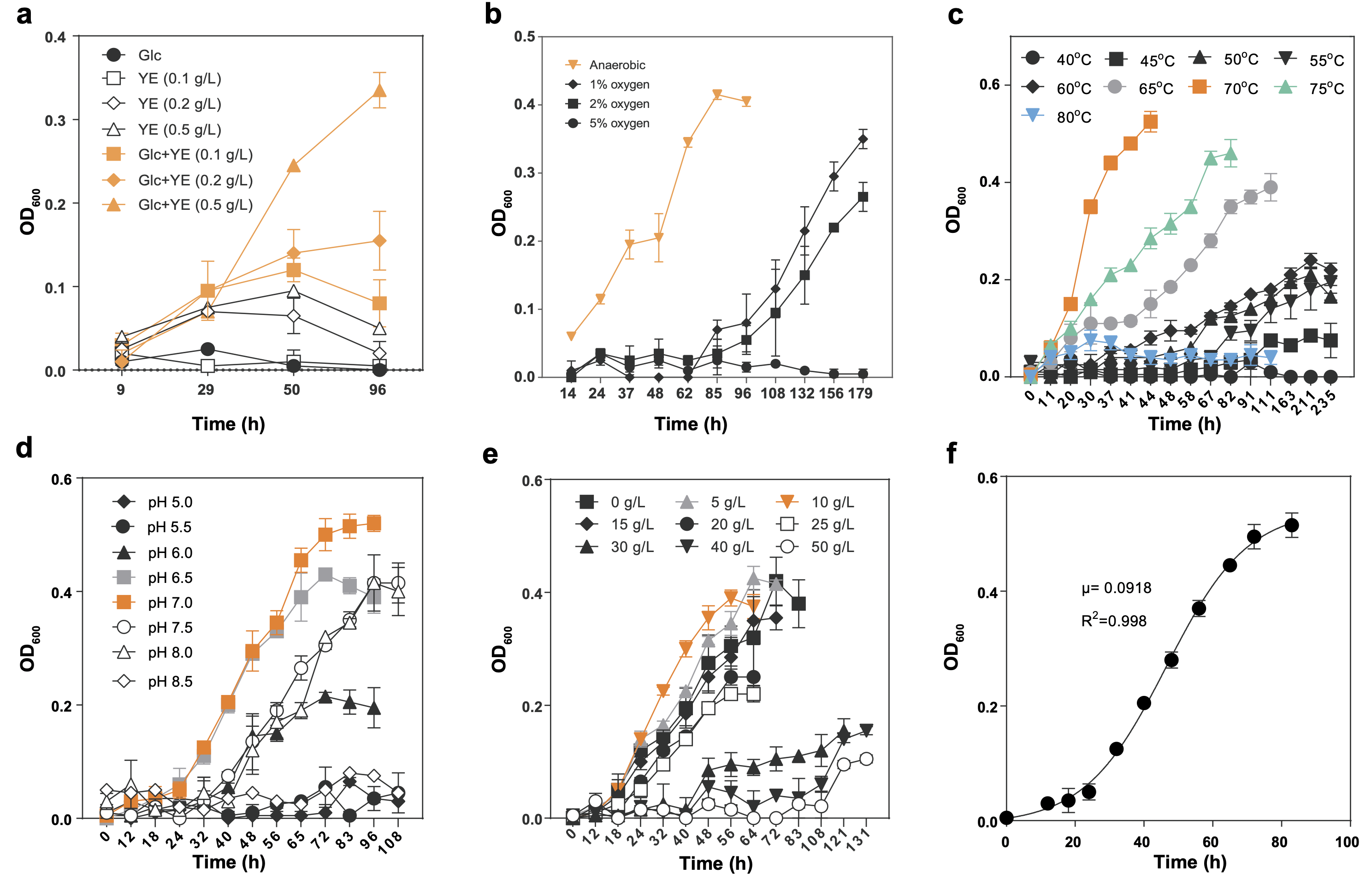


**Fig. S3. Physiological experiments of *Thermatribacter velox* B11^T^. a** Yeast extract (YE) was required for the growth of strain B11^T^ when glucose (glc) used as substrate. **b** Effect of oxygen concentration on growth of strain B11^T^ in non-reduced anaerobic nutrient-rich low salt medium. **c** Effect of temperature on growth of strain B11^T^. **d** Effect of pH on growth of strain B11^T^. **e** Effect of NaCl concentration on growth of strain B11^T^. **f** Logistic growth curve. Fitting the logistic curve based on OD_600_ at optimal conditions (75 ºC, pH 7.0, and 10 g/L) using Logistic 4-PL curve model in GraphPad Prism 7.00 (https://www.graphpad.com). μ, specific growth rate calculated using “Differentiation” analysis with OriginPro 2023 (https://www.originlab.com/).


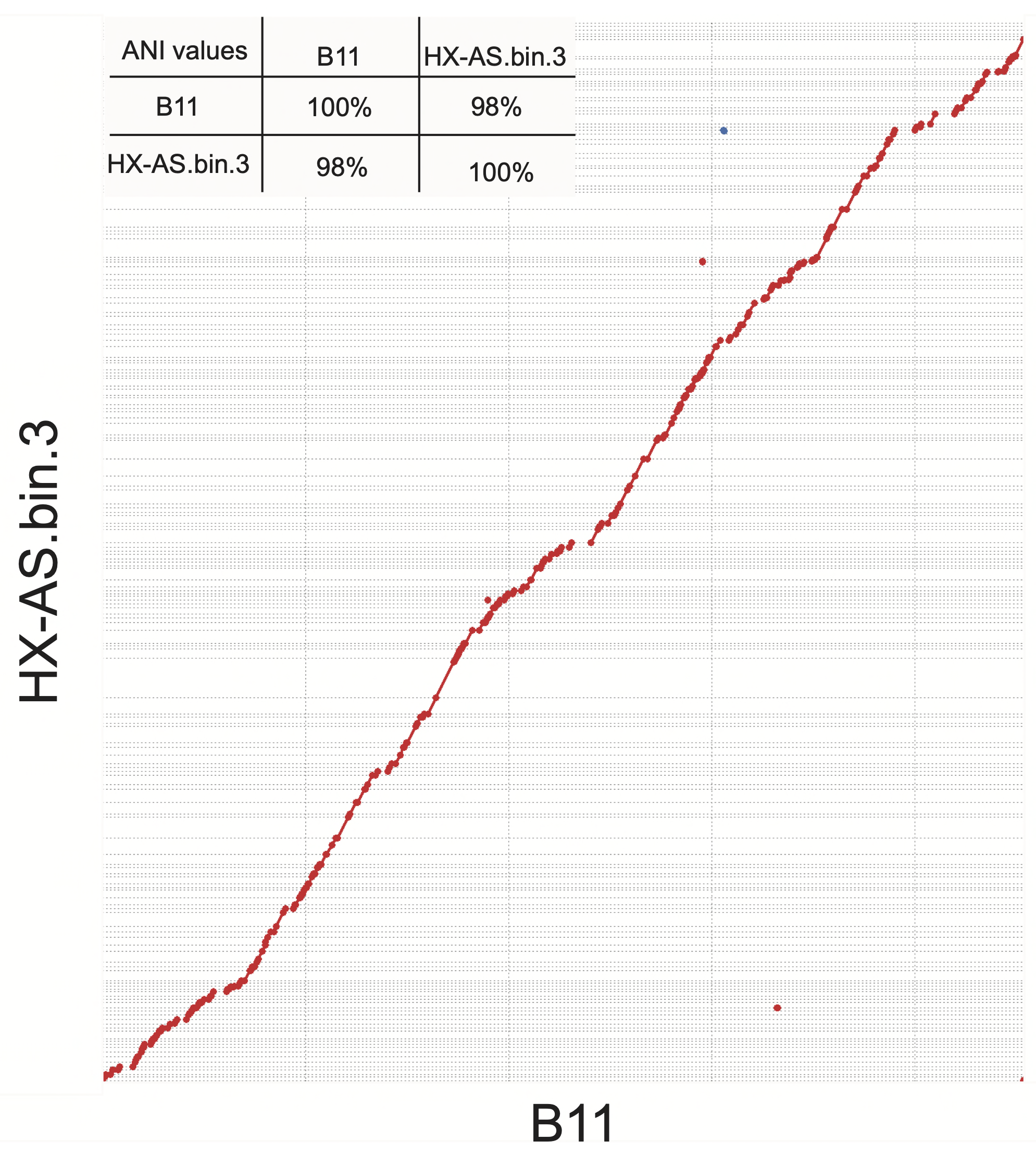


**Fig. S4. Genomic similarity of B11 and HX-AS.bin.3.** The alignment plot of B11 and HX-AS.bin.3 was generated by MUMmer software [1]. The ANI values between B11 and HX-AS.bin.3 were calculated by pyANI [2].


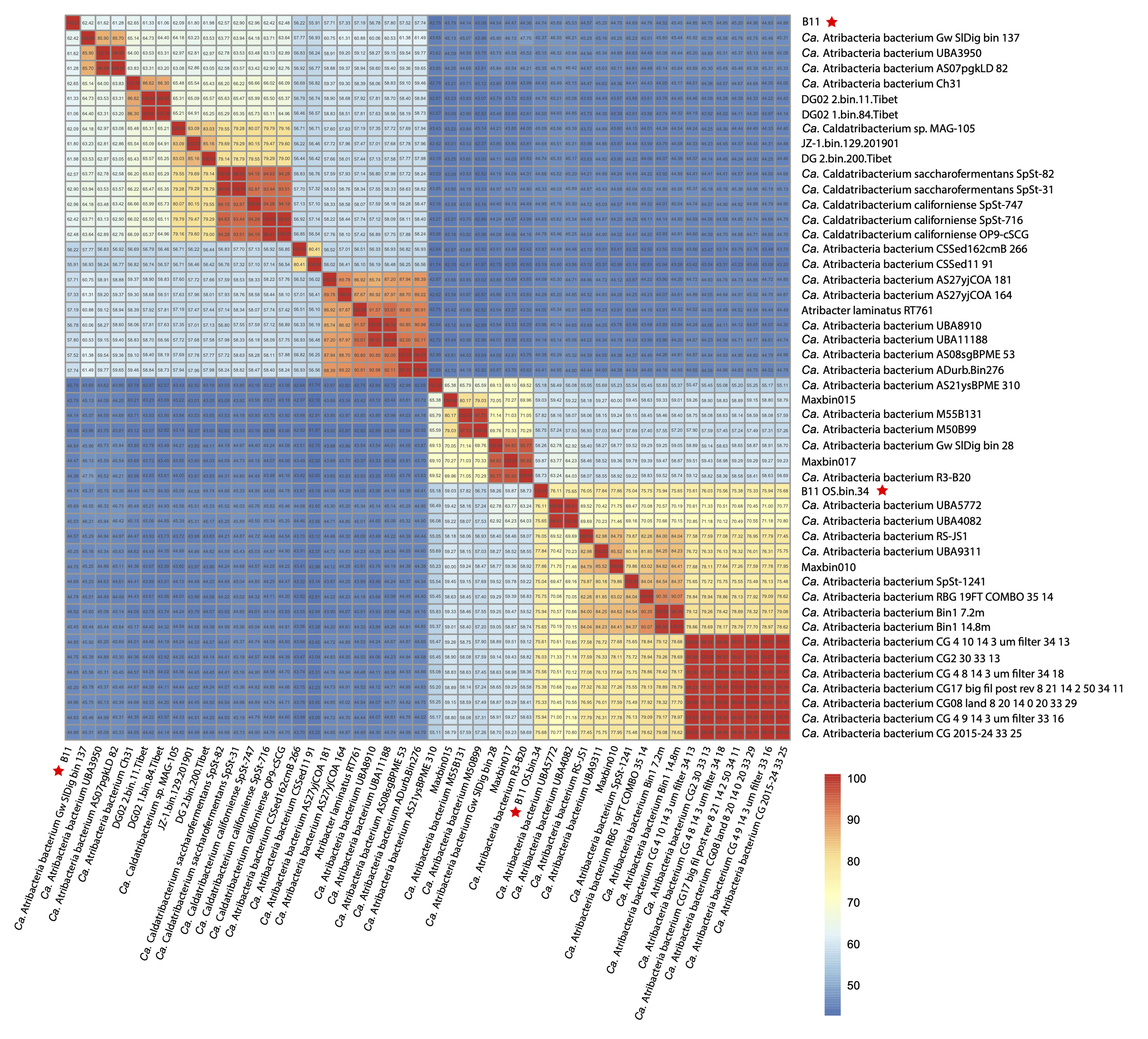


**Fig. S5. Average amino acid identity (AAI) shared among *Atribacterota* genomes.** AAI values were calculated as previous studies [3-5].


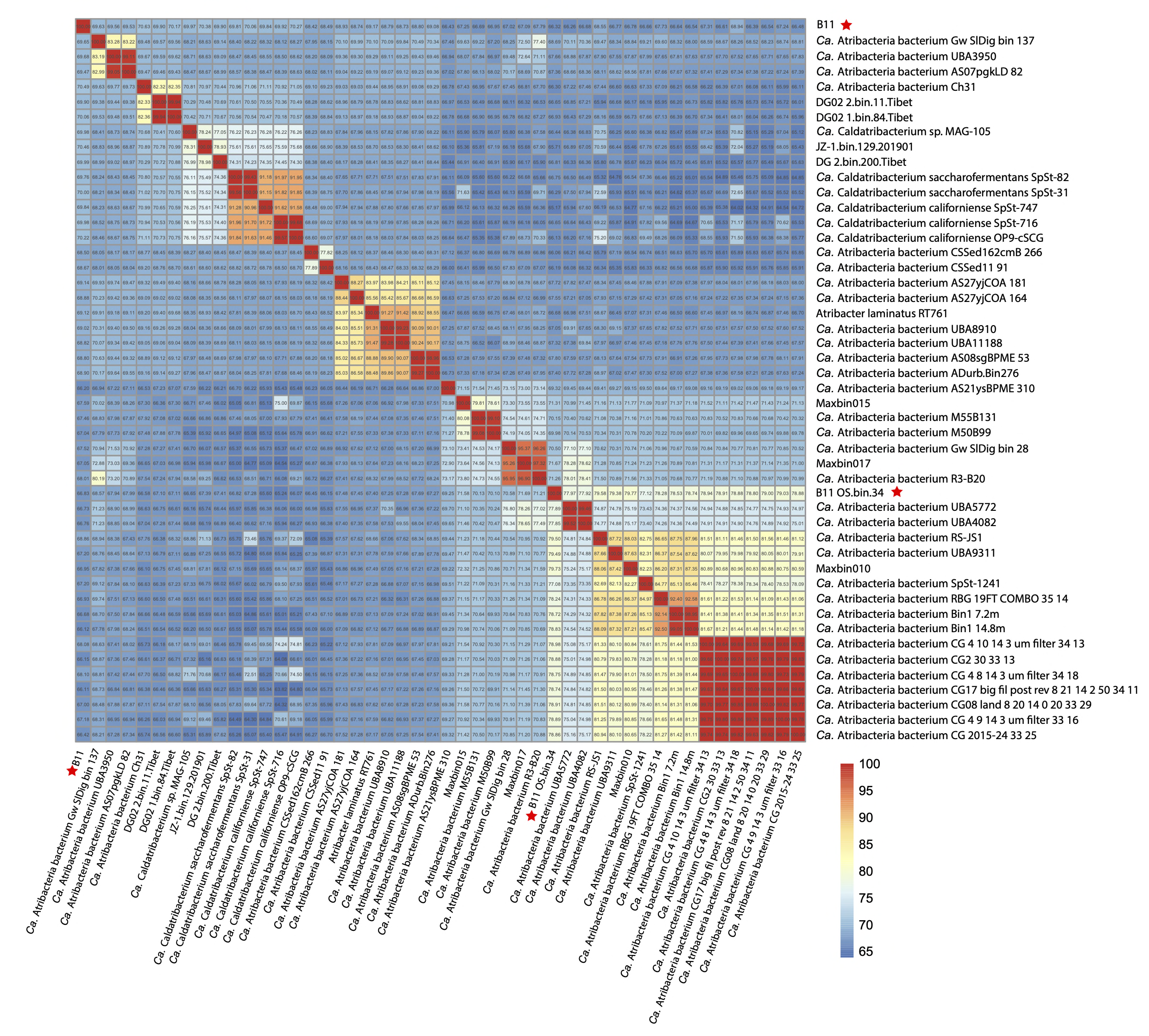


**Fig. S6. Average nucleotide identity (ANI) shared among *Atribacterota* genomes.** ANI values were calculated by pyANI [2].

**
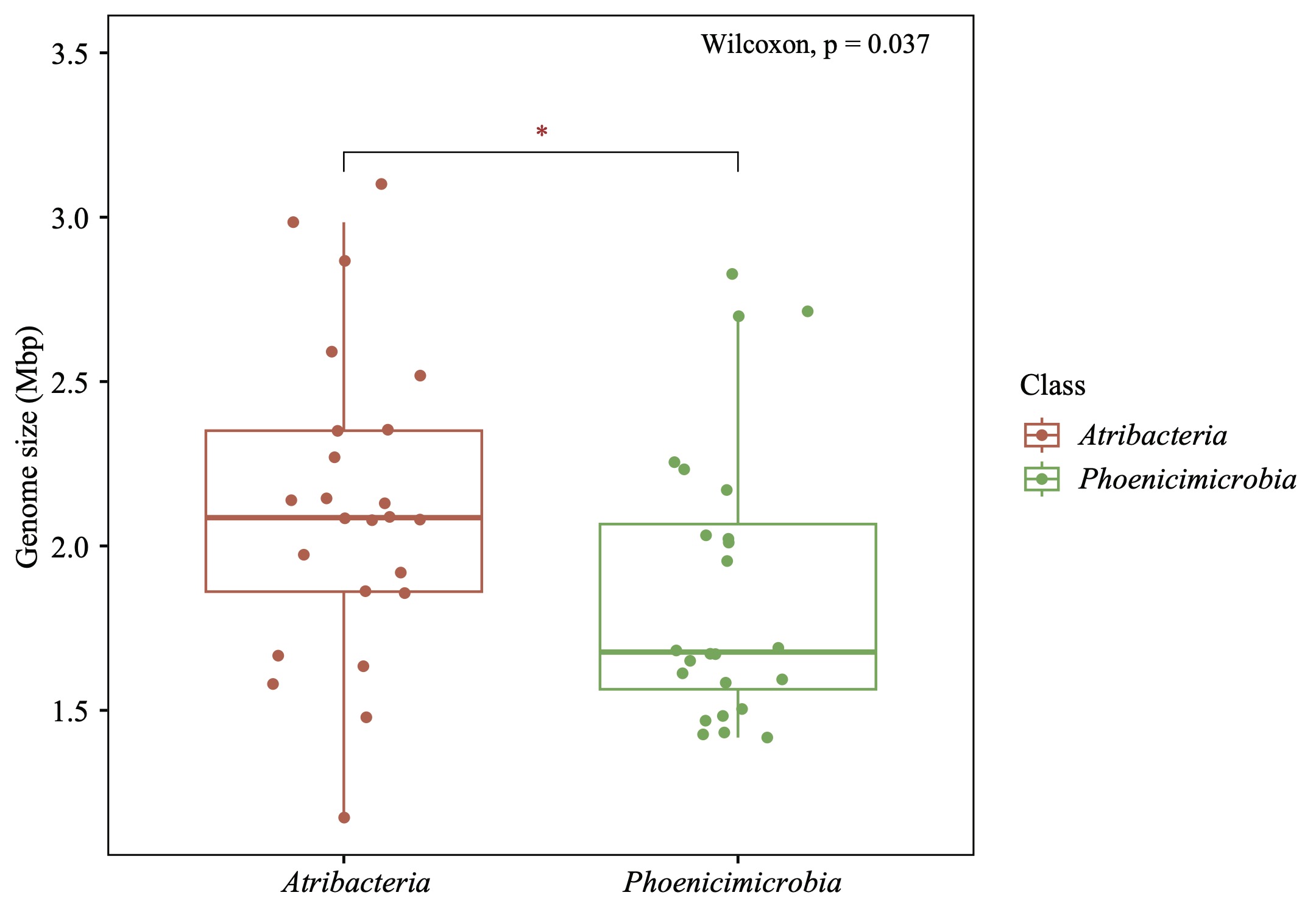
**

**Fig. S7. Difference in assembled genome size of *Atribacteria* and *Phoenicimicrobia*.** The genome size of *Atribacteria* was significantly smaller than the size of *Phoenicimicrobia*. Significant differences among different datasets were denoted by star (P < 0.05).

**
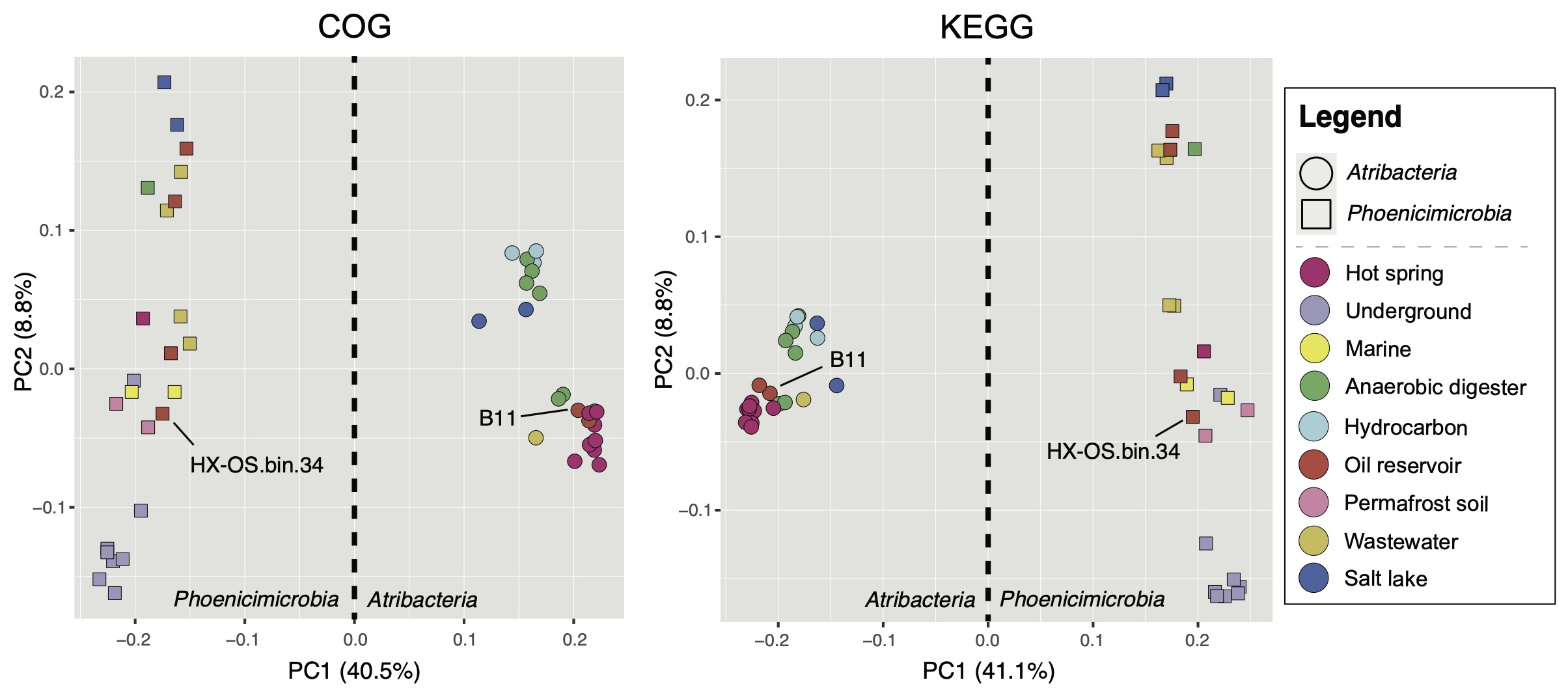
**

**Fig. S8. Principal coordinates analyses (PCoA) based on Clusters of Orthologous Groups (COGs) and KEGG Orthologs (KOs).** The vegan package was used for analysis by using Jaccard method and the ggplot2 package was used for visualization.

**
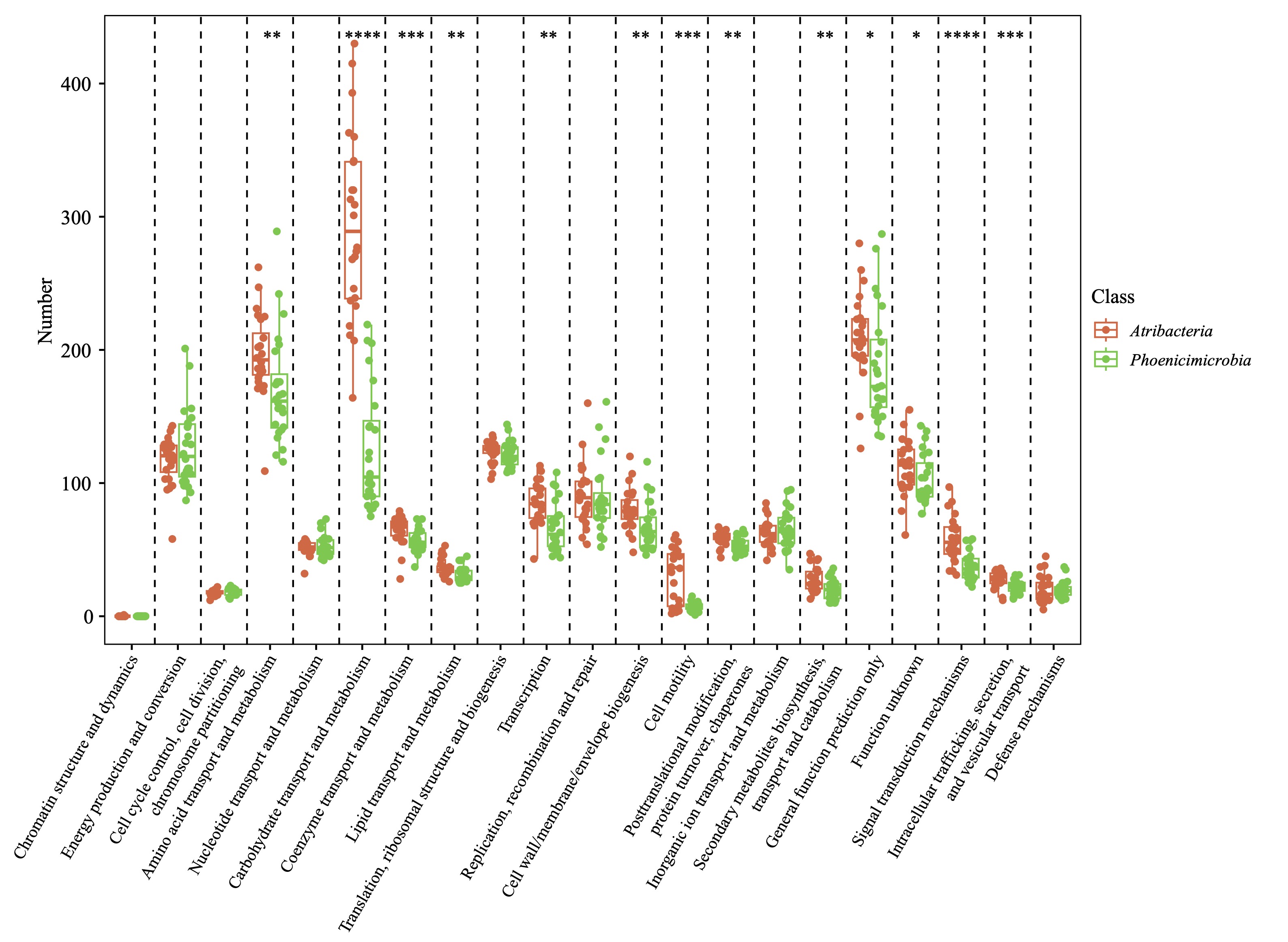
**

**Fig. S9. Clusters of Orthologous Groups (COG) categories of *Atribacteria* and *Phoenicimicrobia*.** Clusters of Orthologous Groups (COG) categories were significantly different between *Atribacteria* and *Phoenicimicrobia*, especially in Carbohydrate transport and metabolism (COG category G), Cell motility (COG category N), Coenzyme transport and metabolism (COG category H), Signal transduction mechanisms (COG category T) and Intracellular trafficking, secretion, and vesicular transport (COG category U). Significant differences among different datasets were denoted by star (P < 0.05).

**
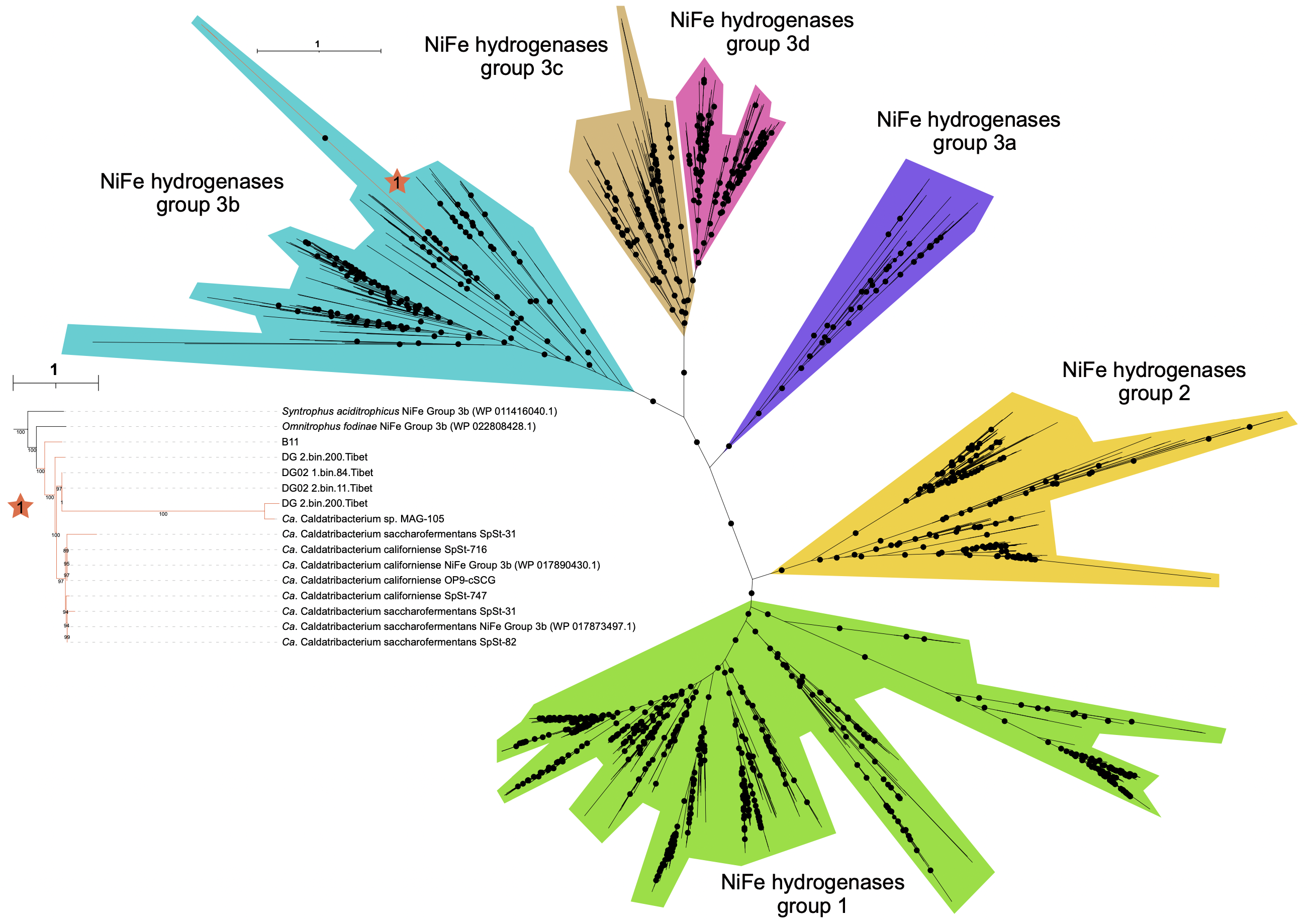
**

**Fig. S10. Phylogenetic tree of groups 1, 2 and 3 [NiFe] hydrogenases catalytic subunits.** Hydrogenase genes of the MAGs recovered in this study are shown with a red star. The reference dataset of hydrogenases was selected from one previous study [6]. Alignments were generated using MUSCLE [7] with 100 iterations and divergent regions were eliminated using TrimAL [8]. The phylogenomic tree was generated using IQ-TREE [9] (v1.6.10; -alrt 1000 -bb 1000 -nt AUTO). The best-fit model (LG+R10) determined by ModelFinder [10] is well supported by Akaike Information Criterion (AIC) and Bayesian Information Criterion (BIC). Bootstrap values > 80% were shown on nodes. Scale bar indicates substitutions per site.

**
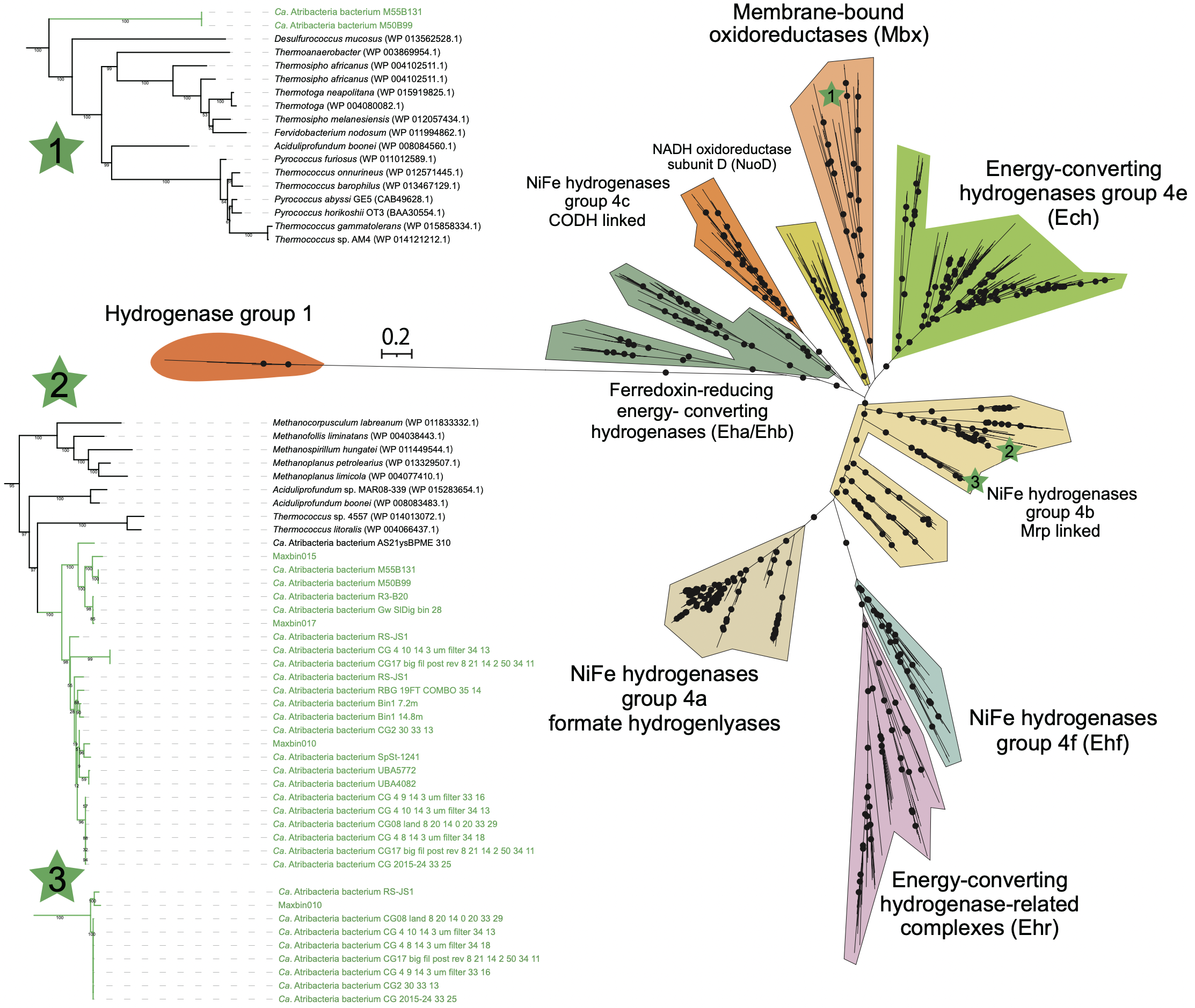
**

**Fig. S11. Phylogenetic tree of [NiFe] hydrogenase group 4 and related complexes.** The reference dataset of hydrogenases was selected from previous studies [6, 11]. Alignments were calculated using MUSCLE [7] with 100 iterations and the divergent regions were eliminated using TrimAL [8]. The IQ-TREE [9] was used for constructing the phylogenomic tree. The best-fit model (LG+R10) determined by ModelFinder [10] is well supported by Akaike Information Criterion (AIC) and Bayesian Information Criterion (BIC). Bootstrap values > 80% were shown on nodes. Sequences of [NiFe] hydrogenase group 1 were used as outgroup. Scale bar indicates substitutions per site.

**
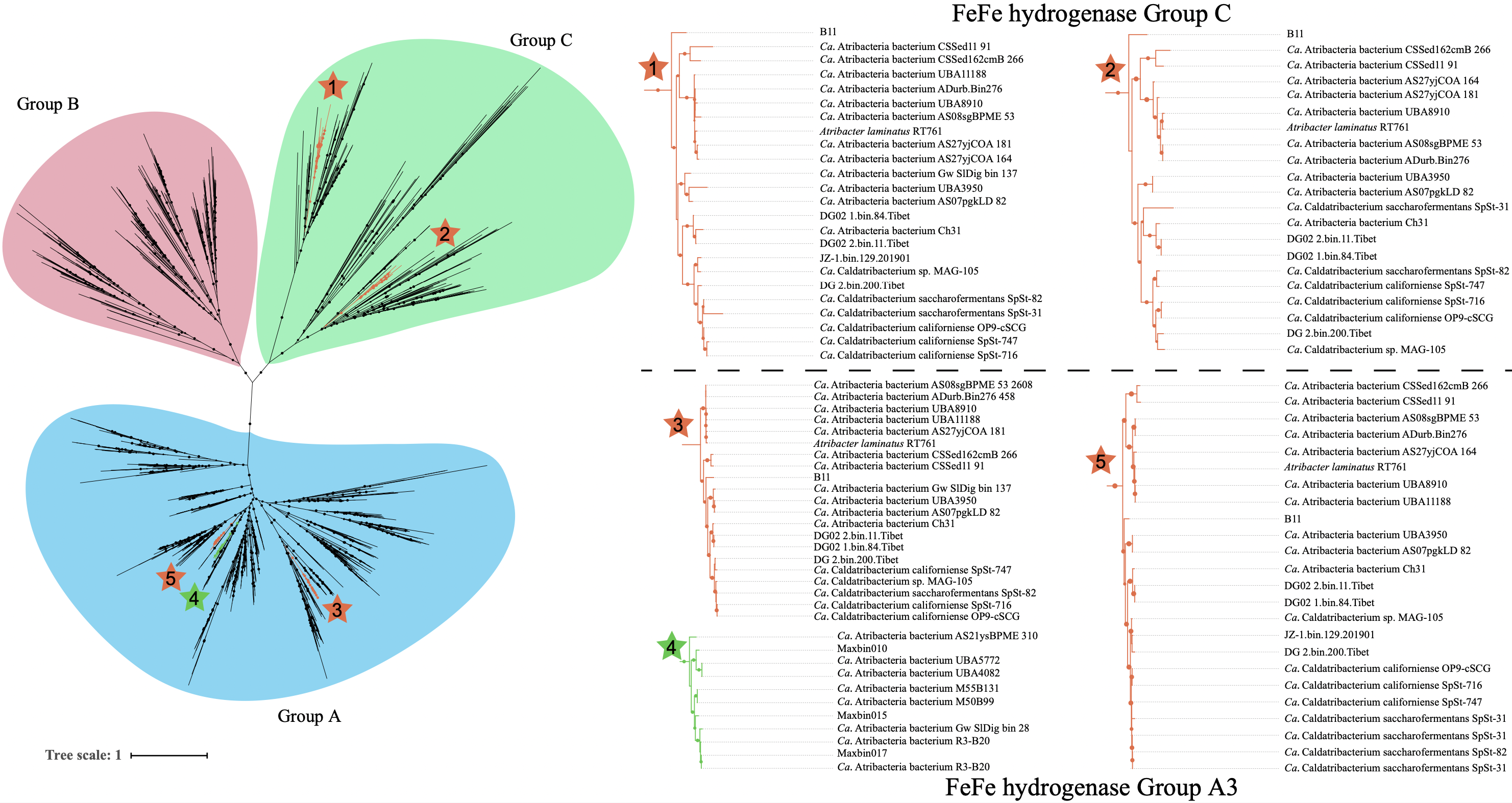
**

**Fig. S12. Phylogenetic tree of [FeFe] hydrogenase.** The reference dataset of hydrogenases was selected from one previous study [6, 11]. Alignments were generated using MUSCLE [7] with 100 iterations and divergent regions were eliminated using TrimAL [8]. The phylogenomic tree was generated using IQ-TREE [9] (v1.6.10; -alrt 1000 -bb 1000 -nt AUTO). The best-fit model (WAG+F+R10) determined by ModelFinder [10] is well supported by Akaike Information Criterion (AIC) and Bayesian Information Criterion (BIC).


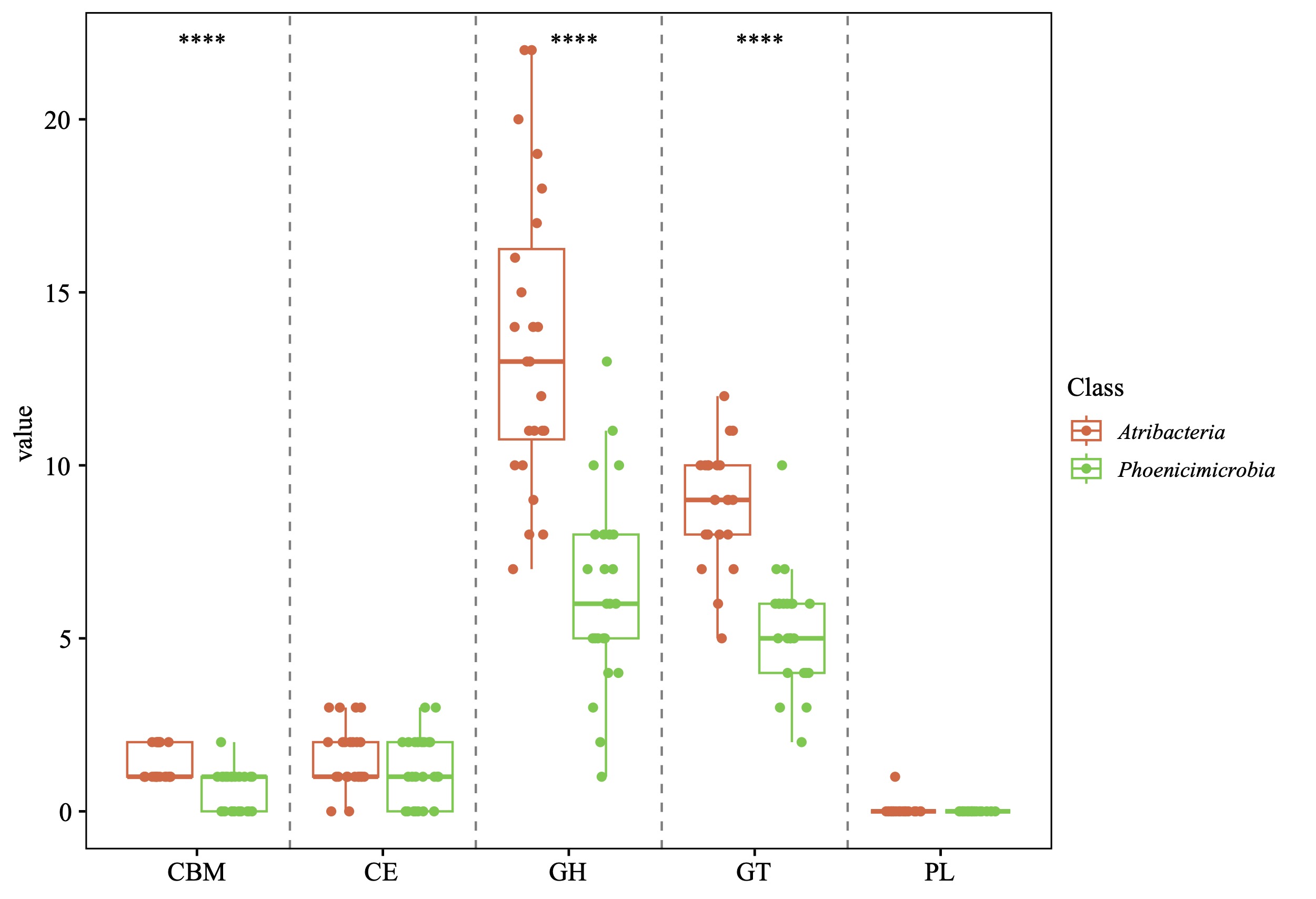


**Fig. S13. Different CAZy families and their distribution in *Atribacteria* and *Phoenicimicrobia*.** CBM: carbohydrate-binding modules. CE: carbohydrate esterase. GH: glycoside hydrolyase. GT: glycosyl transferase. PL: polysaccharide lyase. Significant differences among different datasets were denoted by star (P < 0.05).


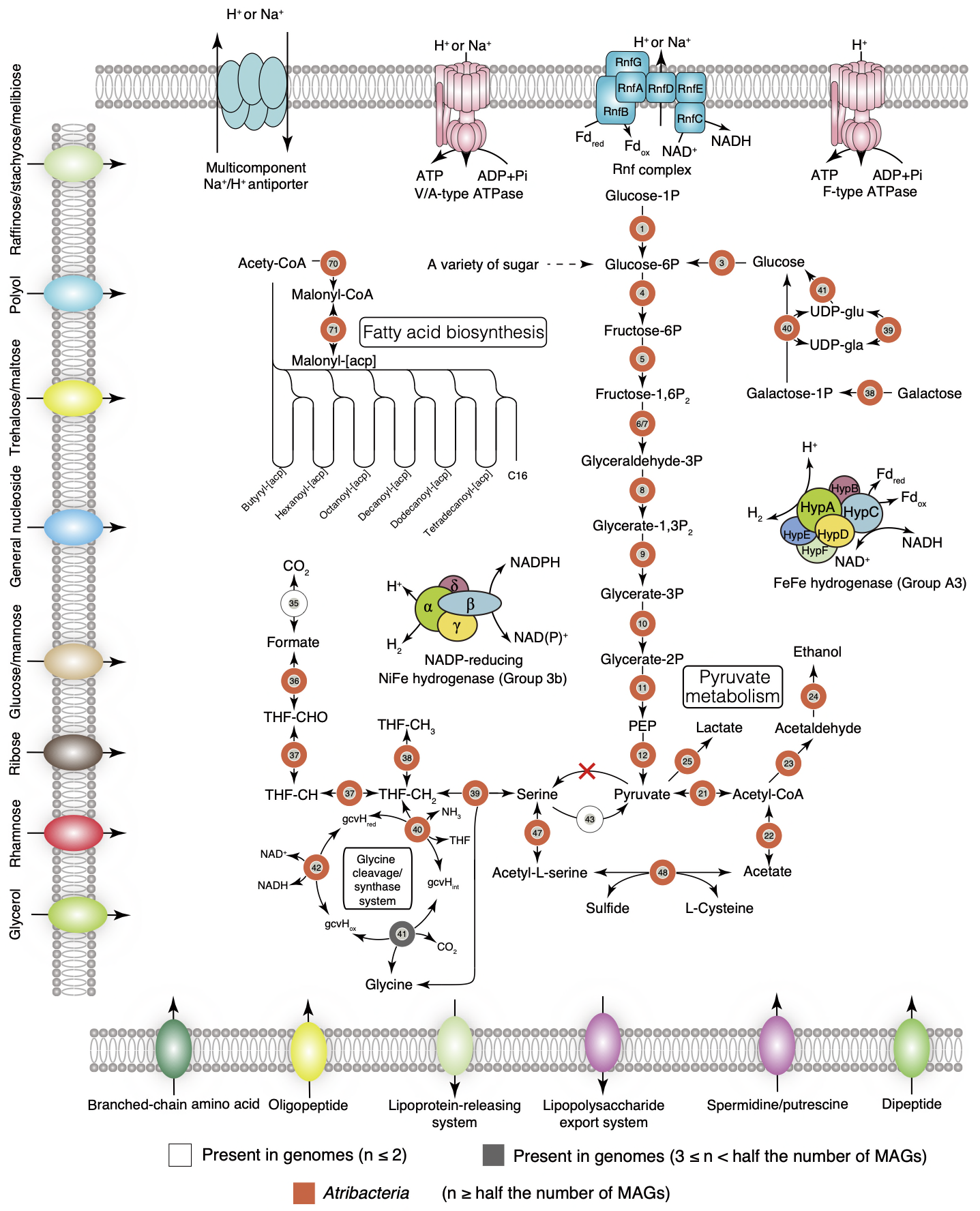


**Fig. S14. Schematic view of genes involved in sugar fermentation of *Atribacteria*.**


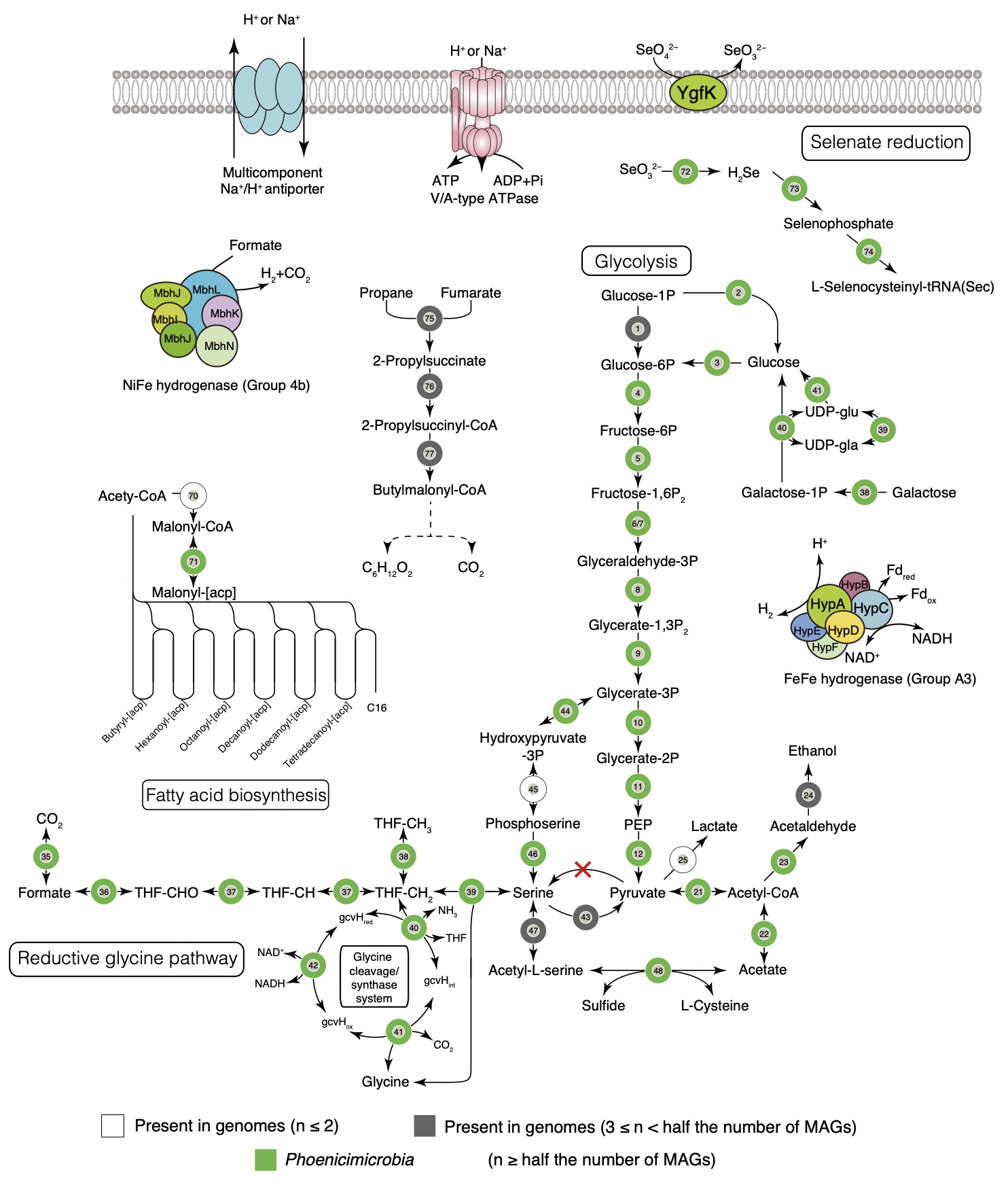


**Fig. S15. Schematic view of genes involved in hydrocarbon metabolism of *Phoenicimicrobiia*.** If the propane as the case, the overall reaction is C_3_H_8_ + C_4_H_4_O_4_ = C_6_H_12_O_2_ + CO_2_, ∆G° = -91.67 (kJ/mol) [12].


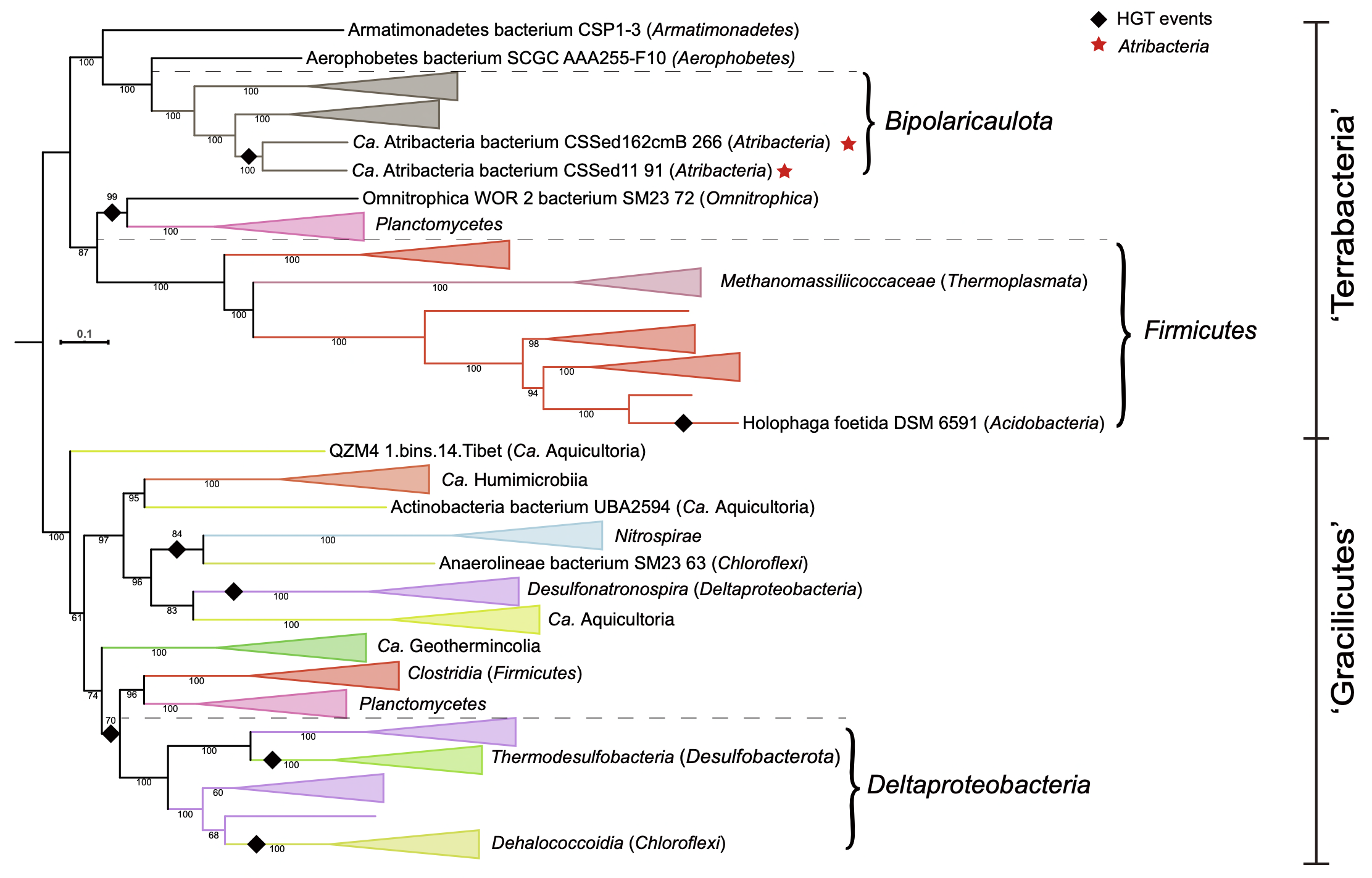


**Fig. S16. Phylogenetic tree based on concatenated AcsAB sequences.** Maximum likelihood phylogeny of concatenated AcsAB sequences was performed as previous studies [3, 13]. The subunits of AcsA and AcsB were aligned using MUSCLE with 100 iterations [7], respectively. The alignments were concatenated by using a perl script (https://github.com/nylander/catfasta2phyml). IQ-Tree [9] was used for phylogenetic inference with parameters (-alrt 1000 -bb 1000 -nt AUTO). The best-fit model was LG+R7, which was well supported by Bayesian Information Criterion (BIC). Phylogenetic tree was visualized and annotated using iTOL [14].


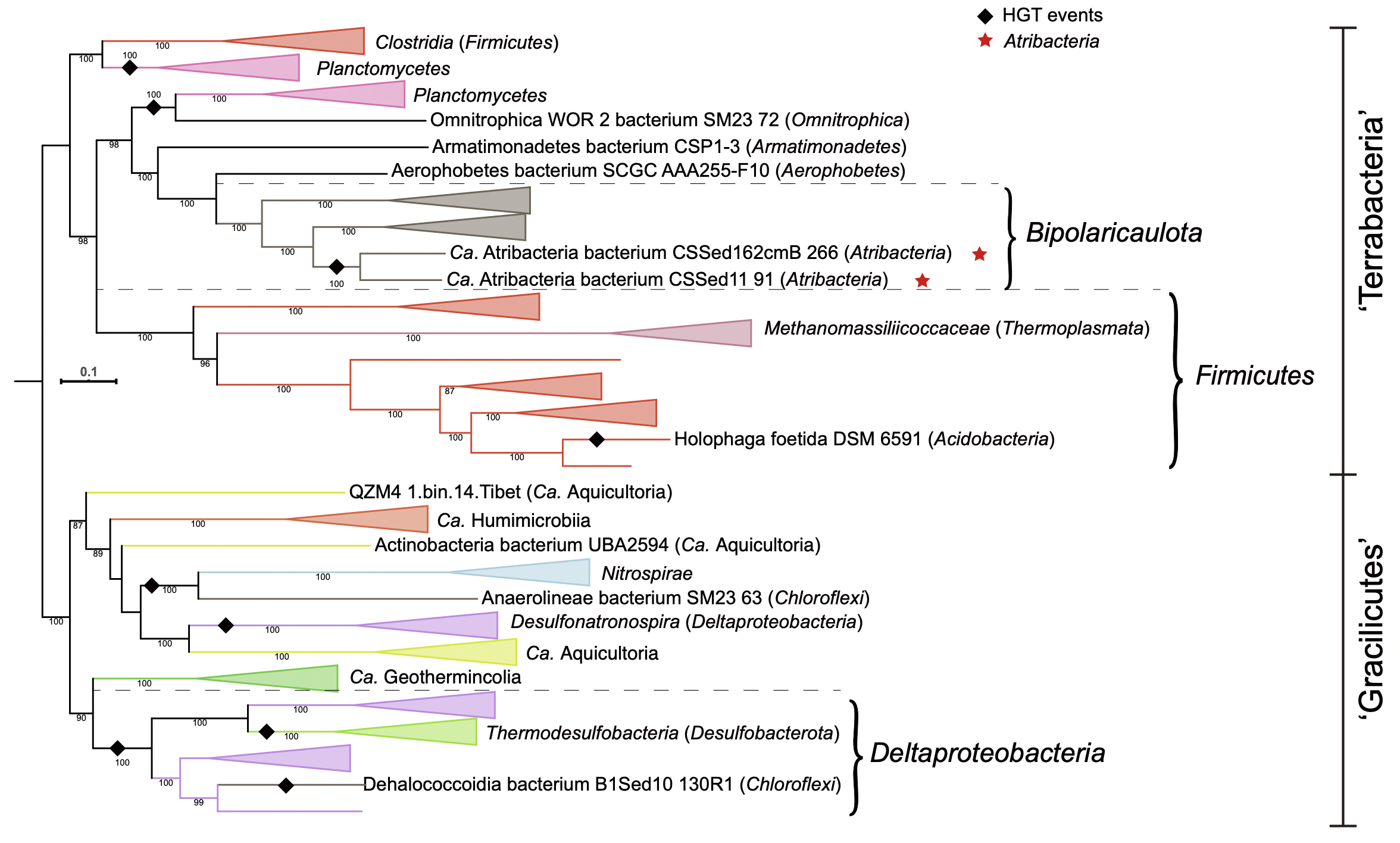


**Fig. S17. Phylogenetic tree based on concatenated AcsABC sequences.** Maximum likelihood phylogeny of concatenated AcsABC sequences was performed as previous studies [3, 13]. The subunits of AcsA, AcsB and AcsC were aligned using MUSCLE with 100 iterations [7], respectively. The alignments were concatenated by using a perl script (https://github.com/nylander/catfasta2phyml). IQ-Tree [9] was used for phylogenetic inference with parameters (-alrt 1000 -bb 1000 -nt AUTO). The best-fit model was LG+F+R8, which was well supported by Akaike Information Criterion (AIC), Corrected Akaike Information Criterion (cAIC) and Bayesian Information Criterion (BIC). Phylogenetic tree was visualized and annotated using iTOL [14].


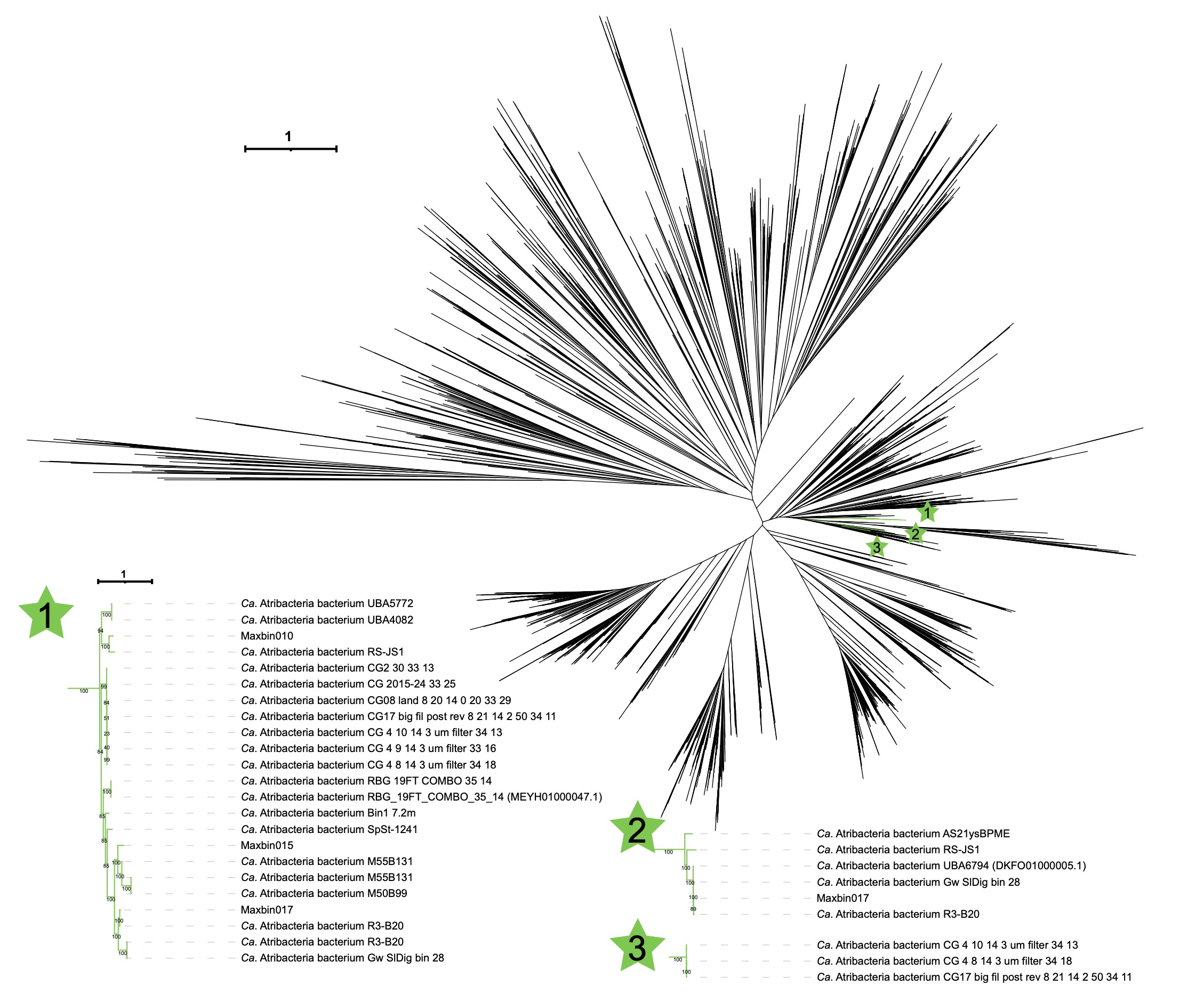


**Fig. S18. Phylogenetic tree of FdhA protein sequences.** The FdhA sequences were aligned using MUSCLE5 [15], and divergent regions were eliminated using TrimAL [8]. The IQ-Tree was used for phylogenetic inference [9], and the best model LG+R10 was well supported by Akaike Information Criterion (AIC), and Bayesian Information Criterion (BIC). Phylogenetic tree was visualized and annotated using iTOL [14].


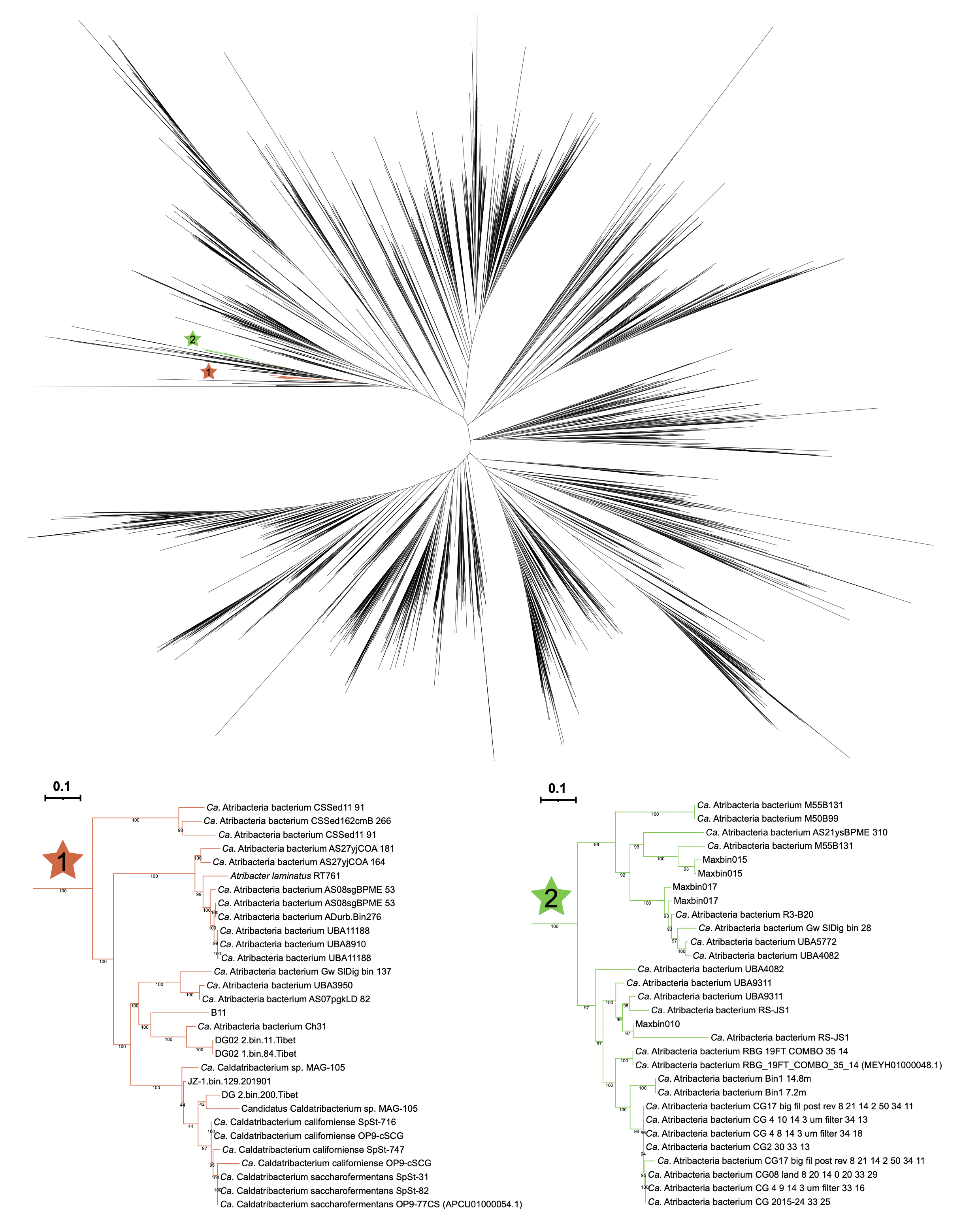


**Fig. S19. Phylogenetic tree of Fhs protein sequences.** The Fhs sequences were aligned using MUSCLE5 [15], and divergent regions were eliminated using TrimAL [8]. The IQ-Tree was used for phylogenetic inference [9], and the best model LG+R10 was well supported by Akaike Information Criterion (AIC), and Bayesian Information Criterion (BIC). Phylogenetic tree was visualized and annotated using iTOL [14].


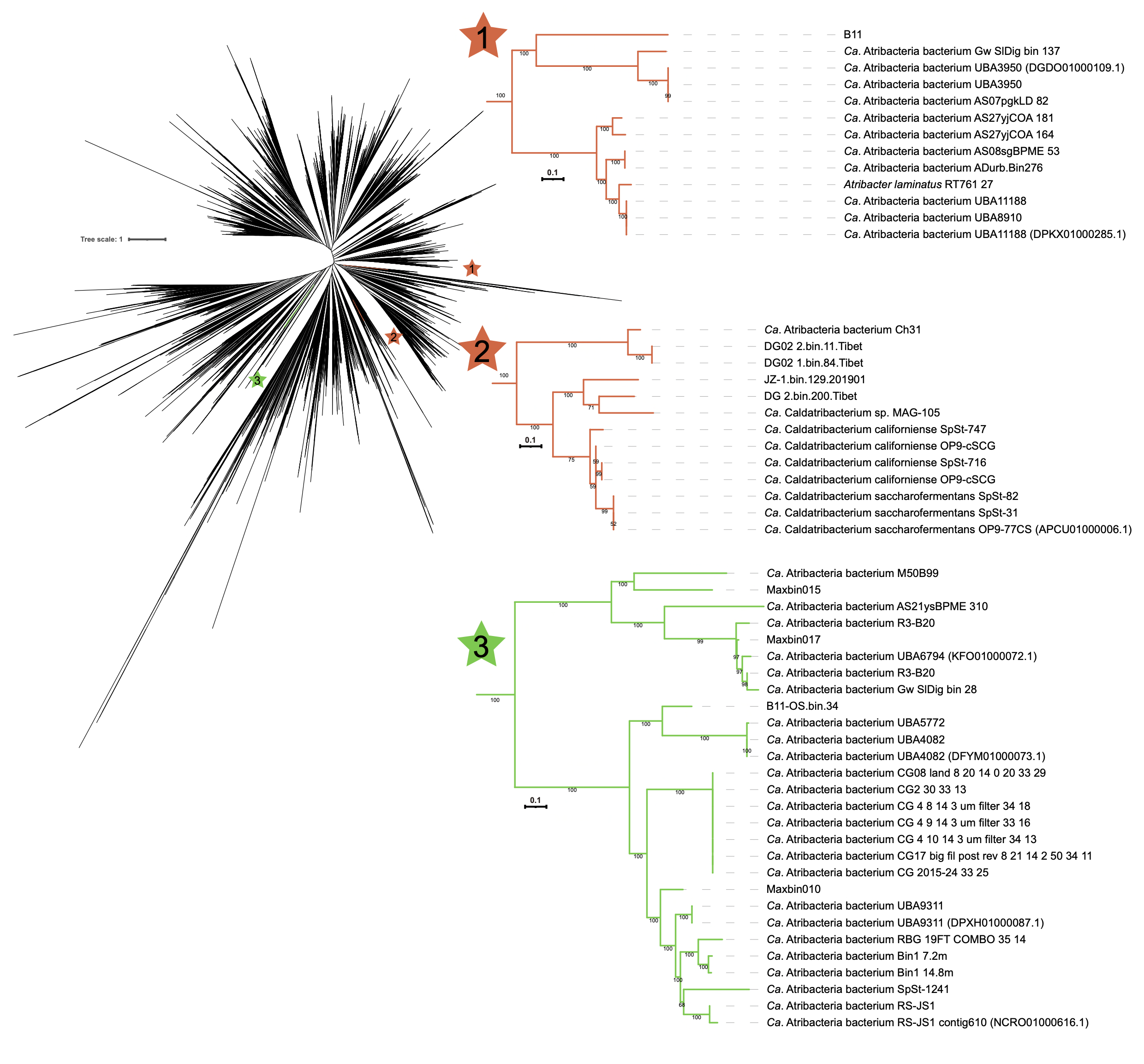


**Fig. S20. Phylogenetic tree of FolD protein sequences.** The FolD sequences were aligned using MUSCLE5 [15], and divergent regions were eliminated using TrimAL [8]. The IQ-Tree was used for phylogenetic inference [9], and the best model LG+R10 was well supported by Akaike Information Criterion (AIC), and Bayesian Information Criterion (BIC). Phylogenetic tree was visualized and annotated using iTOL [14].


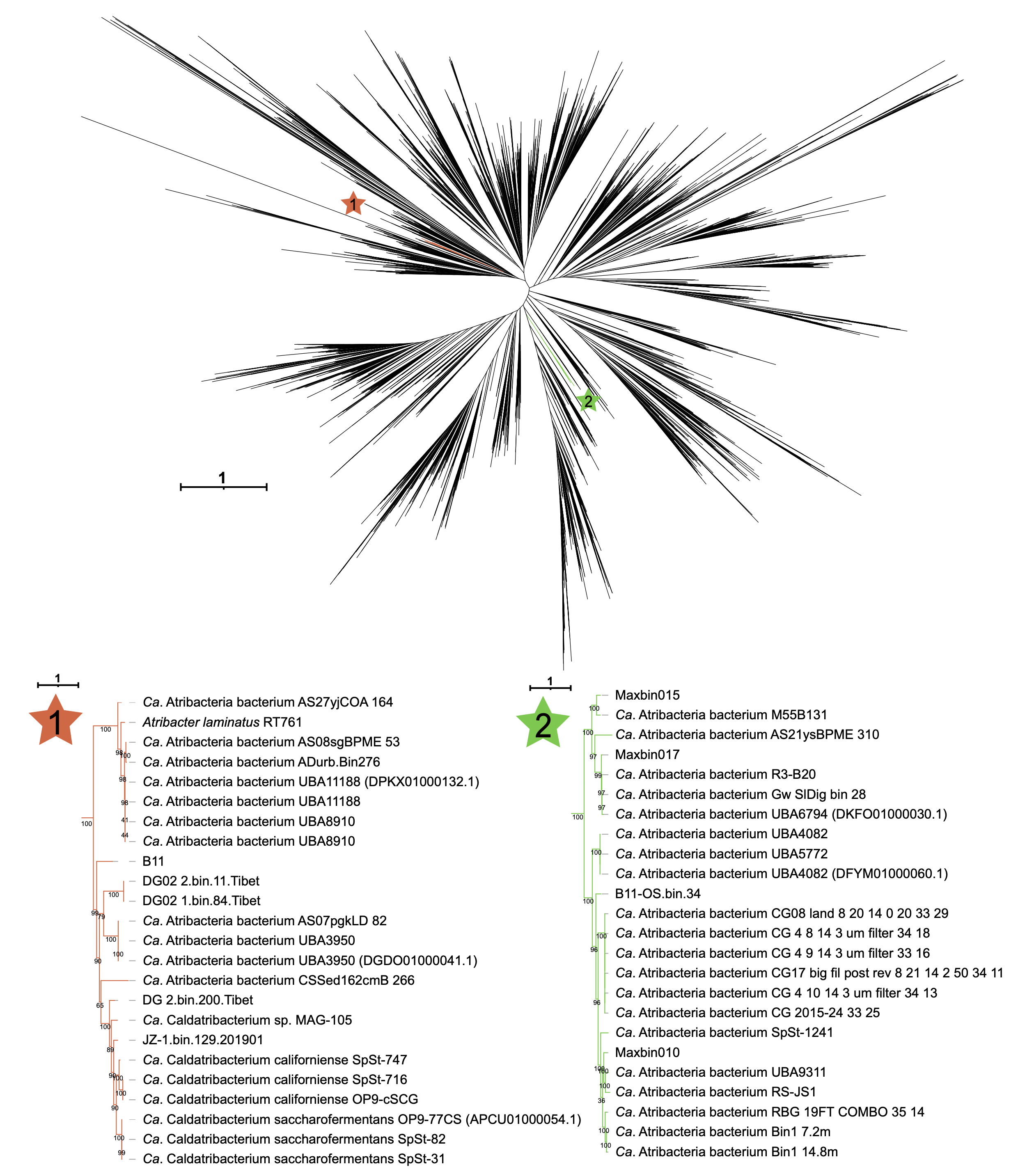


**Fig. S21. Phylogenetic tree of GlyA protein sequences.** The GlyA sequences were aligned using MUSCLE5 [15], and divergent regions were eliminated using TrimAL [8]. The IQ-Tree was used for phylogenetic inference [9], and the best model LG+R10 was well supported by Akaike Information Criterion (AIC), and Bayesian Information Criterion (BIC). Phylogenetic tree was visualized and annotated using iTOL [14].


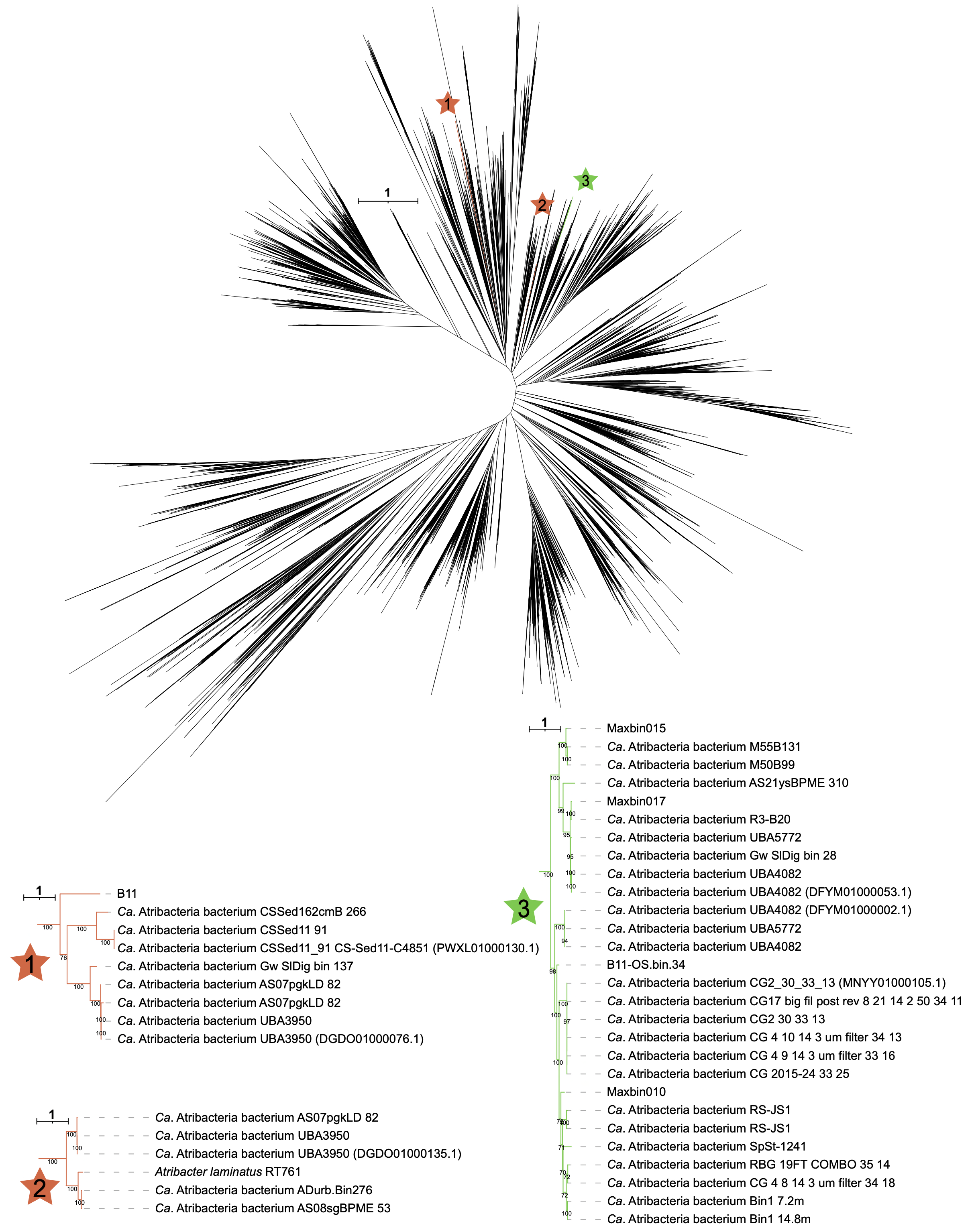


**Fig. S22. Phylogenetic tree of GcvT protein sequences.** The GcvT sequences were aligned using MUSCLE5 [15], and divergent regions were eliminated using TrimAL [8]. The IQ-Tree was used for phylogenetic inference [9], and the best model LG+R10 was well supported by Akaike Information Criterion (AIC), and Bayesian Information Criterion (BIC). Phylogenetic tree was visualized and annotated using iTOL [14].


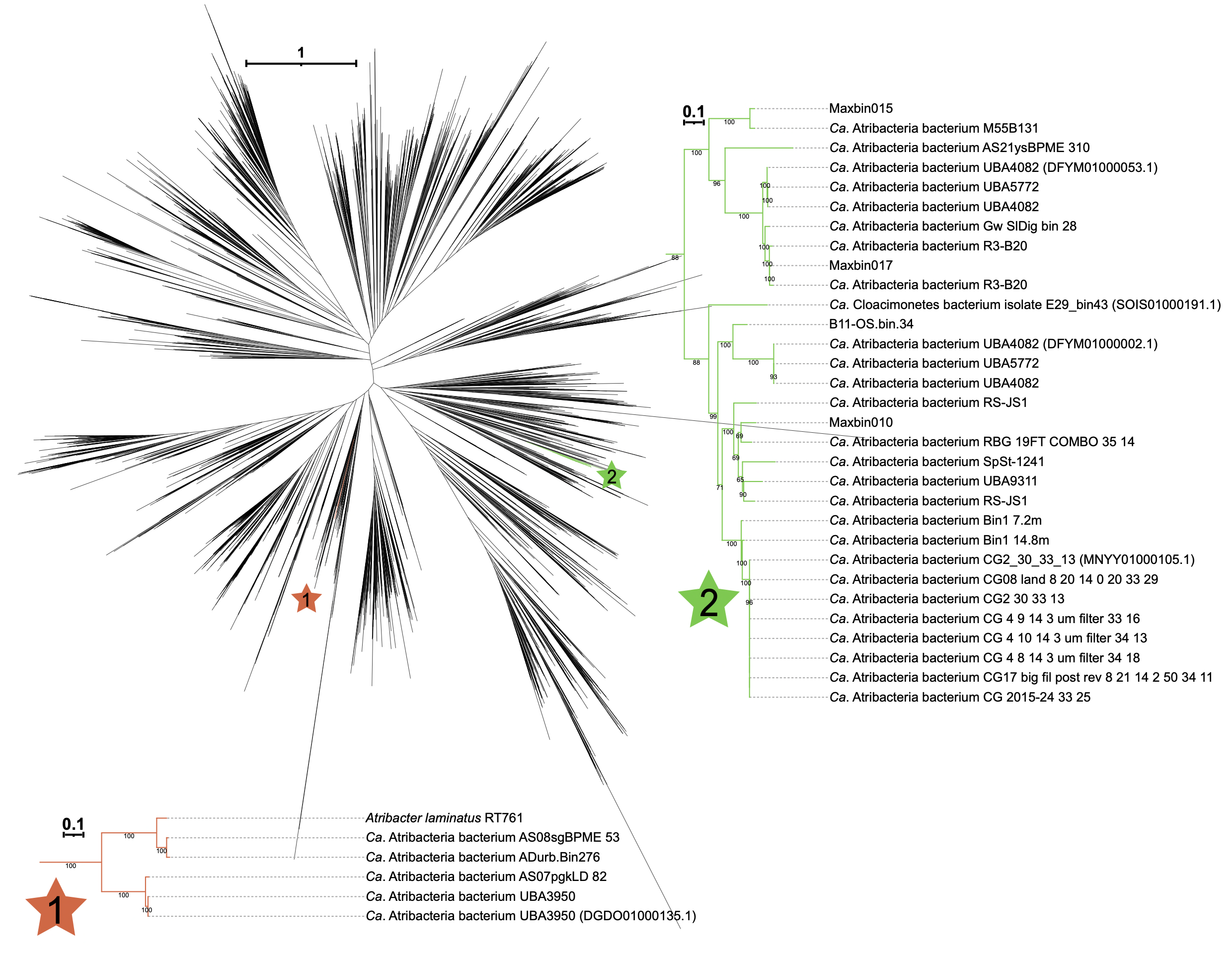


**Fig. S23. Phylogenetic tree of GcvPA protein sequences.** The GcvPA sequences were aligned using MUSCLE5 [15], and divergent regions were eliminated using TrimAL [8]. The IQ-Tree was used for phylogenetic inference [9], and the best model LG+R10 was well supported by Akaike Information Criterion (AIC), and Bayesian Information Criterion (BIC). Phylogenetic tree was visualized and annotated using iTOL [14].


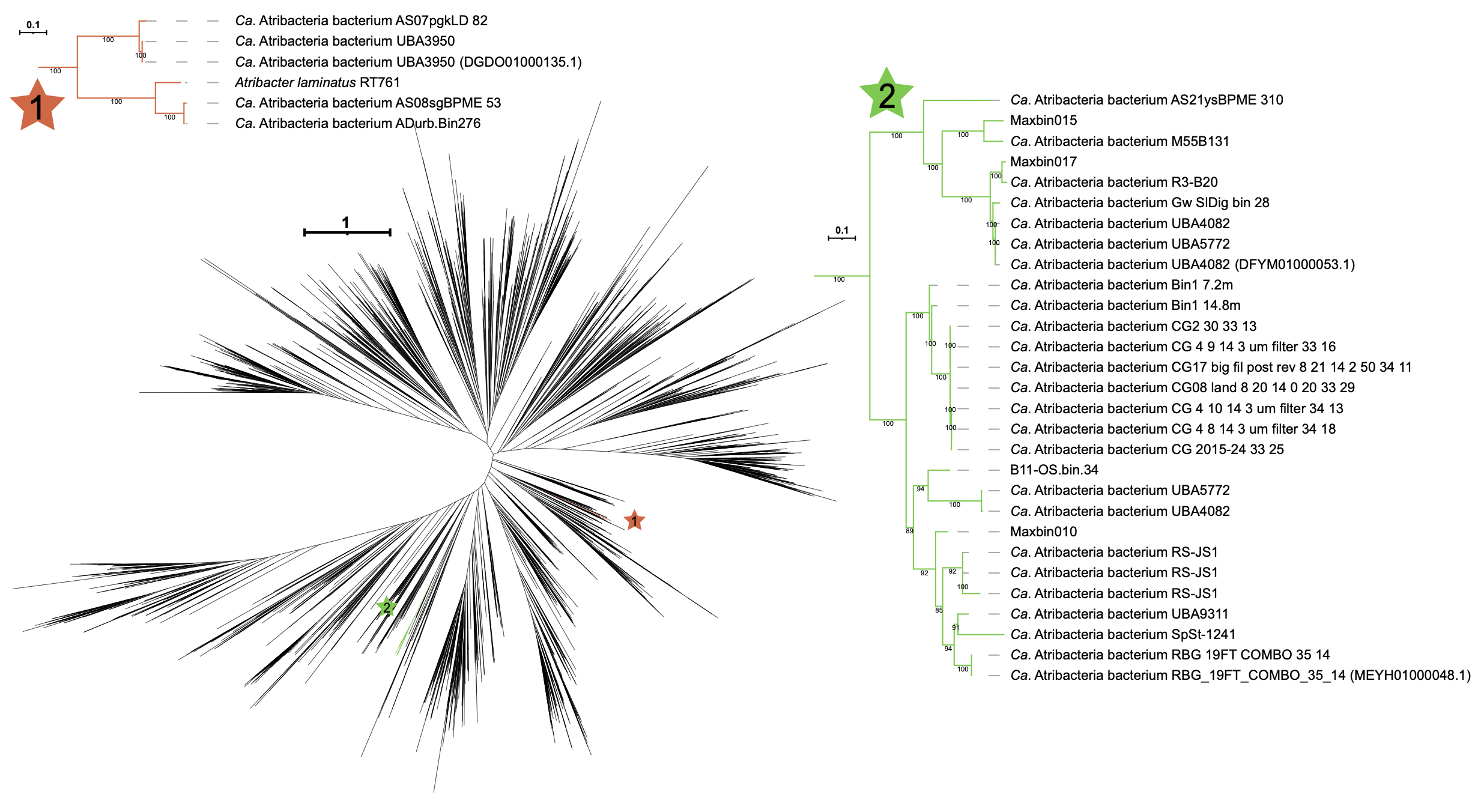


**Fig. S24. Phylogenetic tree of GcvPB protein sequences.** The GcvPB sequences were aligned using MUSCLE5 [15], and divergent regions were eliminated using TrimAL [8]. The IQ-Tree was used for phylogenetic inference [9], and the best model LG+R10 was well supported by Akaike Information Criterion (AIC), and Bayesian Information Criterion (BIC). Phylogenetic tree was visualized and annotated using iTOL [14].


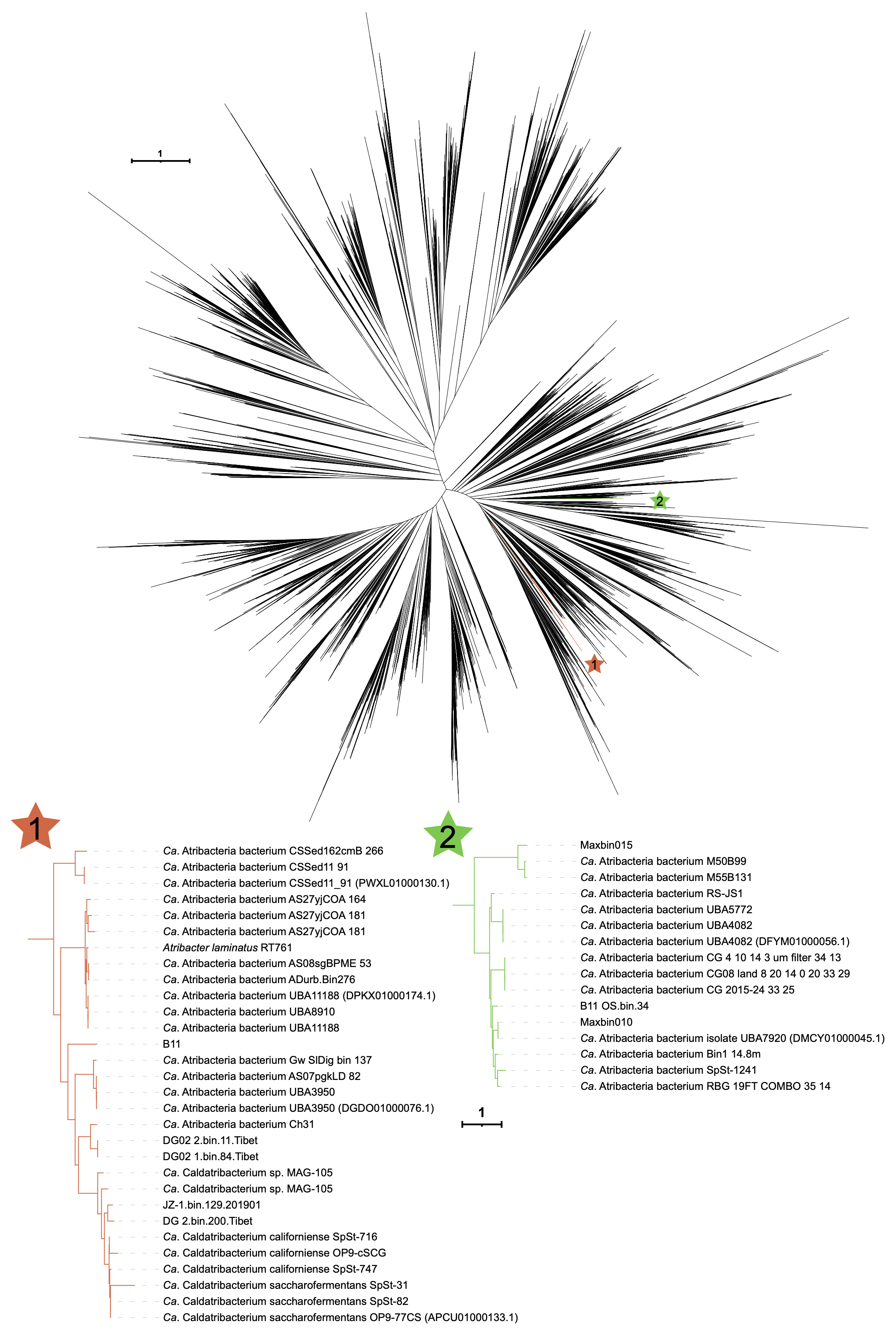


**Fig. S25. Phylogenetic tree of PdhD protein sequences.** The PdhD sequences were aligned using MUSCLE5 [15], and divergent regions were eliminated using TrimAL [8]. The IQ-Tree was used for phylogenetic inference [9], and the best model LG+R10 was well supported by Akaike Information Criterion (AIC), and Bayesian Information Criterion (BIC). Phylogenetic tree was visualized and annotated using iTOL [14].


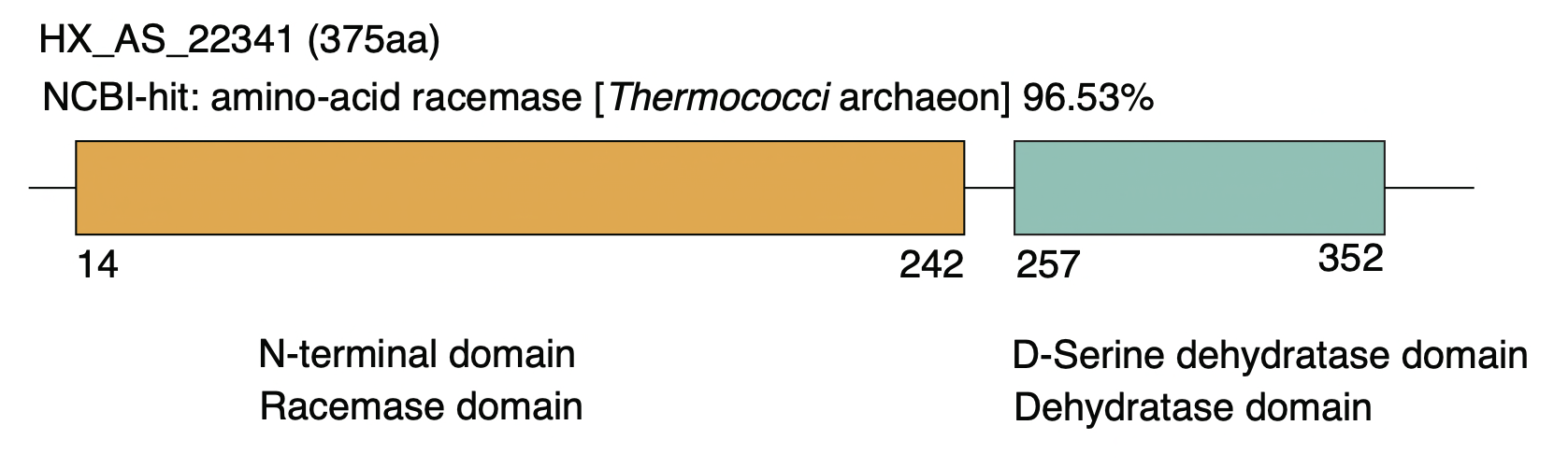


**Fig. S26. D-serine dehydratase domain and racemase domain from the classes *Thermococci*.**

# Supplementary Text

1. Physiology of *Thermatribacter velox* B11^T^.

The isolate B11 was obtained by serial picking and re-streaking isolated white, opaque, circular, and flat colonies that formed after two weeks of incubation on low salt (LS) medium supplemented with 3 mM acetate. B11 is the second culture of the phylum *Atribacterota*. Cells of strain B11 were fusiform rod or ovoid shape with 0.4-0.5 μm in width and 0.6-1.9 μm in length (Fig. 1b-d), Gram-stain negative, and non-motile. The cell envelope consisted of three lipid membrane-like layers (LML) (Fig. 1b), similar to *Atribacter laminatus* RT761^T^ [16]. The inner LML appeared to envelop the nucleoid, likely forming a confined compartment (Fig. 1d). Like *A*. *laminatus* RT761^T^, strain B11 had a large fraction of saturated fatty acids (74.9%) in its membrane lipids (Table S3). A major transition in the composition of *iso*-C_15:0_, C_16:0,_ and C_18:0_ was observed when the growth temperature was increased from 52.2°C to 71/72°C, indicating that these saturated fatty acids contribute to the adaptation of B11 to high temperature.

2. Genomic-based taxonomy of *Atribacterota*.

These 48 MAGs were assigned to two classes based on the phylogenomic tree and GTDB-Tk [17] results (Table S4). One class was known as *Atribacteria*, another one belongs to a novel class (*Phoenicimicrobia*) that was previously known as JS1. There are 31 species identified by cut-off ANI value 95% [18] (Fig. S4). In Class1 (*Atribacteria*), the AAI values between B11 and other genomes are clearly lower than 65% (Fig. S3), which used for defining families [19]. Furthermore, the genome of B11 was assigned to a novel family by GTDB-Tk [17]. Therefore, we proposed that *Thermatribacter velox* B11^T^ should be a novel family, *Thermatribacteraceae* fam. nov., which is clearly different from the first isolate *Atribacter laminatus* RT761^T^. Other taxonomic ranks were determined by GTDB-Tk [17]. Finally, our refined taxonomic classification resulted in 2 classes, 2 orders, 5 families, 13 genera, and 31 species (Table S4).

3. Nomenclature of members of *Atribacterota*.

**Description of *Atribacter fermentans* sp. nov. (S14)**

*Atribacter fermentans* (fer.men´tans. L. part. adj. *fermentans* fermenting, referring to the source of genome from anaerobic digestion of organic wastes).

Type genome: AS27yjCOA_181 (GCA_012517485.1) obtained from the anaerobic digestion of organic wastes.

**Description of *Atribacter alterifermentans* sp. nov. (S15)**

*Atribacter alterifermentans* (al.te.ri.fer.men´tans. L. masc. adj. *alter* another, the other; L. part. adj. *fermentans* fermenting, N.L. masc. adj. *alterifermentans* the other fermenter, referring to the source of genome from anaerobic digestion of organic wastes).

Type genome: AS27yjCOA_164 (GCA_012517635.1), obtained from the anaerobic digestion of organic wastes.

**Description of *Atribacter hydrocarboniphilus* sp. nov. (S17)**

*Atribacter hydrocarboniphilus* (hy.dro.car.bo.ni´phi.lus. N.L. neut. n. *hydrocarbonum* hydrocarbon; Gr. masc. adj. *philos* loving; N.L. masc. n. *hydrocarboniphilus* hydrocarbon loving, referring to the source of the metagenomic sample).

Type genome: UBA11188 (GCA_003542295.1), obtained from a hydrocarbon metagenome assembly.

**Description of *Atribacter allofermentans* sp. nov. (S18)**

*Atribacter allofermentans* (al.lo.fer.men´tans. Gr. masc. adj. *allos*, other; L. part. adj. *fermentans* fermenting, N.L. masc. adj. *allofermentans* another fermenter, referring to the source of genome from anaerobic digestion of waste samples).

Type material: ADurb.Bin276 (GCA_002069605.1), obtained from a metagenome assembly of samples containing mixtures of municipal and industrial waste sludge and sewage.

**Description of *Nitricultor* gen. nov. (G5)**

*Nitricultor* (Nit.ri.cul´tor. L. neut. n. *nitrum* soda; L. masc. n. *cultor* an inhabitant; N.L. masc. n. *Nitricultor* an inhabitant of the soda environment).

Type species: *Nitricultor lacus*.

**Description of *Nitricultor lacus* sp. nov. (S12)**

*Nitricultor lacus* (la´cus. L. gen. n. *lacus* of a lake).

Type genome: CSSed162cmB_266 (GCA_007128145.1), obtained from the metagenome assembly from a hypersaline soda lake sediment in Siberia.

**Description of *Nitricultor siberiensis* sp. nov. (S13)**

*Nitricultor siberiensis* (si.be.ri.en´sis. N.L. masc. adj. *siberiensis* pertaining to Siberia, the source of the metagenomic sample).

Type genome: CSSed11_91 (GCA_003561145.1), obtained from the metagenome assembly from a hypersaline soda lake sediment in Siberia.

**Description of *Thermatribacter* gen. nov. (G1)**

*Thermatribacter* (Therm.a.tri.bac´ter. Gr. masc. adj. *thermos* hot; N.L. masc. n. *Atribacter* a bacterial genus; N.L. masc. n. *Thermatribacter* a thermophilic *Atribacter*).

Anaerobic, extremely thermophilic, Gram-negative, non-motile, and non-spore-forming fusiform rod or ovoid shaped cells. Major cellular fatty acids are C_16:0_, C_18:0_ and *iso*-C_15:0_. Acetate, ethanol, hydrogen and carbon dioxide are produced from glucose.

Type species: *Thermatribacter velox*.

**Description of *Thermatribacter velox* sp. nov. (S1)**

*Thermatribacter velox* (ve´lox. L. masc. adj. *velox* rapid).

Shows the following characteristics in addition to those given for the genus: colonies are white, opaque, circular, flat colony with entire margin and 2–5 mm in diameter. Cells have a size of 0.4 – 0.5 μm in width and 0.6 –1.9 μm in length. No flagellum is observed. Grows at 45-75ºC, at pH 6.0-7.6 and in the presence of 0-40 g/L NaCl, with the optimal growth at 70ºC, pH 6.5-7.0 with 10 g/L NaCl. Yeast extract is required for growth. Growth occurs with hexose (ribose, xylose), pentose (glucose, galatose, frucose, and trehalose), alditol (mannitol), disaccharide (lactose), and polysaccharide (xylan), and weakly use fumarate.

The type strain B11^T^ (=CCAM 969^T^=JCM 39351^T^) was isolated from an oil sludge collected from an oil tank of Shengli Oilfield in China.

**Description of *Thermatribacteraceae* fam. nov. (F1)**

*Thermatribacteraceae* (Therm.a.tri.bac.te.ra.ce´ae. N.L. masc. n. *Thermatribacter* a bacterial genus; -*aceae*, ending to denote a family; N.L. pl. fem. n. *Thermatribacteraceae* the *Thermatribacter* family).

The description is same as that of the genus *Thermatribacter*.

Type genus: *Thermatribacter*.

**Description of *Caldatribacterium oleiphilum* sp. nov. (S6)**

*Caldatribacterium oleiphilum* (o.le.i´phi.lum L. neut. n. *oleum* oil; N.L. neut. adj. suff. ­*-philum* loving; N.L. neut. n. *oleiphilum* oil-loving, referring to the source of the genome from an oil field).

Type genome: MAG-105 (CGA_014359405.1), obtained from the metagenome assembly of Shengli oil field at Shandong province.

**Description of *Caldatribacterium thermophilum* sp. nov. (S7)**

*Caldatribacterium thermophilum* (ther.mo´phi.lum. Gr. fem. adj. *thermê* heat; N.L. neut. adj. suff. *-philum* loving; N.L. neut. adj. *thermophilum* heat loving).

Type genome: JZ-1.bins.129.201901 (PRJNA970932) obtained from the metagenome assembly of a hot spring sample.

**Description of *Caldatribacterium caloriphilum* sp. nov. (S8)**

*Caldatribacterium caloriphilum* (ca.lo.ri´phi.lum. L. masc. n. *calor* heat; N.L. neut. adj. suff. *-philum* loving; N.L. neut. adj. *caloriphilum* heat loving).

Type genome: DG_2.bin.200.Tibet (PRJNA970932) obtained from the metagenome assembly of a hot spring sample.

**Description of *Caldatribacterium caldum* sp. nov. (S10)**

*Caldatribacterium caldum* (cal´dum. L. neut. adj. *caldum*, hot, pertaining to the source of the metagenome from hot spring).

Type material: SpSt-747 (IMG Genome ID: 3300009503) obtained from the metagenome assembly from a hot spring sample.

**Description of *Sordicultor* gen. nov. (G2)**

*Sordicultor* (Sor.di.cul´tor. L. fem. n. *sordes* dirt; L. masc. n. *cultor* an inhabitant; N.L. masc. n. *Sordicultor* inhabiting in dirt, referring to the source of genome from wastewater).

Type species: *Sordicultor aquaticus*.

**Description of *Sordicultor aquaticus* sp. nov. (S2)**

*Sordicultor aquaticus* (a.qua´ti.cus. L. masc. adj. *aquaticus* living or growing by water).

Type genome: Gw_SIDig_bin_137 (GCA_018056825.1), obtained from the metagenome assembly of a waste water.

**Description of *Sordicultor fermentans* sp. nov. (S3)**

*Sordicultor fermentans* (fer.men´tans. L. masc. adj. *fermentans* fermenting, referring to the source of genome from anaerobic digestion of organic wastes).

Type genome: UBA3950, obtained from the anaerobic digestion of organic wastes.

**Description of *Profundicultor* gen. nov. (G3)**

*Profundicola* (Pro.fun.di´co.la. L. neut. adj. *profundum* deep; L. masc. n. *cultor* an inhabitant; N.L. masc. n. *Profundicultor* inhabitant of the deep, referring to the isolation of the genome from deep water)

Type genome: *Profundicultor thermophilums* .

**Description of *Profundic****ultor* ***aquiphilus* sp. nov. (S4)**

*Profundicola aquiphilus* (a.qui'phi.lus. L. fem. n. *aqua* water; N.L. masc. adj. suff -*philus* loving; N.L. masc. adj. *aquiphilus* water loving)

Type genome: Ch31 (GCA_013314795.1), obtained from a metagenome assembly of sample from deep subsurface aquifer.

**Description of *Profundic****ultor* ***thermophilus* sp. nov. (S5)**

*Profundicola thermophilus* (ther.mo'philus. N.L. masc. adj. *thermê* heat; N.L. masc. adj. suff *-philum* loving; N.L. masc. adj. *thermophilums* heat loving).

Type genome: DG02_1.bin.11.Tibet (PRJNA970932), obtained from a metagenome assembly of hot spring samples from Tengchong, P.R. China.

**Description of *Phoenicimicrobium* gen. nov. (G10)**

*Phoenicimicrobium* (Phoe.ni.ci.mi.cro’bi.um. L. masc. n. *phoenix*, Phoenix, N.L. neut. n. *microbium*, a microbe; N.L. neut. n. *Phoenicimicrobium* a microbe of Phoenix (a mythological symbol of ancient Egypt), referring to as an immortal or resurrected microbe).

Type species: *Pheonicimicrobium oleiphilum*.

**Description of *Phoenicimicrobium oleiphilum* (S23)**

*Phoenicimicrobium oleiphilum* (o.le.i´phi.lum L. neut. n. *oleum* oil; N.L. neut. adj. suff. *-philum* loving; N.L. neut. n. *oleiphilum* oil-loving, referring to the source of the genome from an oil reservoir).

Type genome: HX_OS.bin.34 (PRJNA970932), obtained from the metagenome assembly of an oil reservoir sample.

**Description of *Immundihabitans* gen. nov. (G11)**

*Immundihabitans* (Im.mun.di.ha’bi.tans. L. masc. adj. *immundus* unclean, impure; L. pres. part. *habitans* inhabating; N.L. masc. n. *Immundihabitans* living in dirty habitat).

Type species: *Immundihabitans aquiphilus*.

**Description of *Immundihabitans aquiphilus* sp. nov. (S24)**

*Immundihabitans aquiphilus* (a.qui’phi.lus. L. fem. n. *aqua* water; N.L. masc. adj. suff *-philus* loving, N.L. masc. adj. *aquiphilus* water loving).

Type genome: UBA4082 (GCA_002383355.1), obtained from a metagenome assembly of waste water.

**Description of *Sediminicultor* gen. nov. (G12)**

*Sediminicultor* (Se.di.mi.ni.cul’tor. L. neut. n. *sedimen -inis* sediment; L. masc. n. *cultor* an inhabitant; N.L. masc. n. *Sediminicultor* an inhabitant of sediment).

Type genome: *Sediminicultor quartus*

**Description of *Sediminicultor primus* sp. nov. (S27)**

*Sediminicultor primus* (pri’mus. L. masc. adj. *primus* first, to represent the first genome bin from of the genus).

Type genome: Maxbin010 (PRJNA970932), obtained from a metagenome assembly of an oil reservoir.

**Description of *Sediminicultor secundus* sp. nov. (S25)**

*Sediminicultor secundus* (se.cun’dus. L. masc. adj. *secundus* second, to represent the second genome bin of the genus).

Type genome: RS-JS1 (GCA_002849045.2), obtained from a metagenome assembly of a marine sediment sample.

**Description of *Sediminicultor tertius* sp. nov. (S26)**

*Sediminicultor tertius* (ter’ti.us. L. masc. adj. *tertius* third, to represent the third genome bin of the genus).

Type genome: UBA9311 (GCA_003527405.1), obtained from a metagenome assembly of a marine sediment sample.

**Description of *Sediminicultor quartus* sp. nov. (S28)**

*Sediminicultor quartus* (qu.ar’tus. L. masc. adj. *quartus* fourth, to represent the fourth genome bin of the genus).

Type genome: SpSt-1241 (GCA_011056645.1), obtained from a metagenome assembly of a hot spring sample.

**Description of *Sediminicultor quintus* sp. nov. (S29)**

*Sediminicultor quintus* (quin’tus. L. masc. adj. *quintus* fifth, to represent the fifth genome bin of the genus).

Type genome: RBG_19FT_COMBO_35_14 (GCA_001773955.1) obtained from a metagenome assembly of a Rifle background sediment sample.

**Description of *Sediminicultor sextus* sp. nov. (S30)**

*Sediminicultor sextus* (sex’tus. L. masc. adj. *sextus* sixth, to represent the sixth genome bin of the genus).

Type genome: Bin1_7.2m (GCA_014894735.1), obtained from a metagenome assembly of an ancient permafrost soil sample.

**Description of *Infernicultor* gen. nov. (G13)**

*Infernicultor* (In.fer.ni.cul’tor. L. masc. adj. *infernus* under, below; L. masc. n. *cultor* an inhabitant; N.L. masc. n. *Infernicultor* an inhabitant from below (the ground)).

Type species: *Infernicultor aquiphilus*

**Description of *Infernicultor aquiphilus* sp. nov. (S31)**

*Infernicultor aquiphilus* (a.qui’phi.lus. L. fem. n. *aqua* water; N.L. masc. adj. suff *-philus* loving, N.L. masc. adj. *aquiphilus* water loving).

Type genome: CG2 30 33 13 (GCA_001873345.1), obtained from a metagenome assembly of groundwater.

**Description of *Phoenicimicrobiaceae* fam. nov. (F5)**

*Phoenicimicrobiaceae* (Phoe.ni.ci.mic.ro.bi.a.ce’ae. N.L. neut. n. *Phoenicimicrobium* type genus of the family; L. suff. –*aceae* ending to denote a family; N.L. fem. pl. n. *Phoenicimicrobiaceae* the *Phoenicimicrobium* family).

The family at present contains four genera *Phoenicimicrobium* gen. nov., *Immunidihabitans* gen. nov., *Sediminicultor* gen. nov. and *Infernicultor* gen. nov.

Type genus: *Phoenicimicrobium*.

**Description of *Stramentimicrobium* gen. nov. (G7)**

*Stramentimicrobium* (Stra.men.ti.mi.cro’bi.um. L. neut. n. *stramentum* litter; N.L. neut. n. *microbium* a microbe; N.L. neut. n. *Stramentimicrobium* a microbe from litter).

Type species: *Stramentimicrobium fermentans*.

**Description of *Stramentimicrobium fermentans* sp. nov. (S19)**

*Stramentimicrobium fermentans* (fer.men´tans. L. neut. adj. *fermentans* fermenting, referring to the source of genome from anaerobic digestion of organic wastes).

Type genome: AS27yjBPME_310 (GCA_012520575.1) obtained from the anaerobic digestion of organic wastes.

**Description of *Oleincola* gen. nov. (G8)**

*Oleincola* (O.le.in’co.la. L. neut. n. *oleum* oil; L. masc./fem. n. *incola* inhabitant; N.L. masc. n. *Oleincola* an inhabitant of oil).

Type species: *Oleincola* ***secundus*** .

**Description of *Oleincola primus* sp. nov. (S20)**

*Oleincola primus* (pri’mus. L. masc. adj. *primus* first, to represent the first genome bin from of the genus).

Type genome: Maxbin015 (PRJNA970932), obtained from the metagenome assembly of an oil reservoir.

**Description of *Oleincola secundus* sp. nov. (S21)**

*Oleincola secundus* (se.cun’dus. L. masc. adj. *secundus* second, to represent the second genome bin from of the genus).

Type genome: M50B99 (GCA_018400815.1), obtained from the metagenome assembly of a sample collected from 5.0 m chemocline layer of meromictic lake.

**Description of *Oleihabitans* gen. nov. (G9)**

*Oleihabitans* (O.lei.ha’bi.tans. L. neut n. *oleum* oil; L. pres. part *habitans* an inhabitant; N.L. masc. n. *Oleihabitans* an inhabitant of oil).

Type species: *Oleihabitans oleiphilus*.

**Description of *Oleihabitans oleiphilus* sp. nov. (S22)**

*Oleihabitans oleiphilus* (o.le.i’phi.lus. L. neut. n. *oleum* oil; Gr. masc. adj. *philos* loving; N.L. masc. adj. *oleiphilus* oil-loving, referring to the extraction of the genome from an oil reservoir).

Type genome: Maxbin017 (PRJNA970932), obtained from the metagenome assembly of an oil reservoir.

**Description of *Stramentimicrobiaceae* fam. nov. (F4)**

*Stramentimicrobiaceae* (Stra.men.ti.mi.cro.bi.a.ce’ae. N.L. neut. n. *Stramentimicrobium* type genus of the family; L. suff. –*aceae* ending to denote a family; N.L. fem. pl. n. *Stramentimicrobiaceae* the family of the genus *Stramentimicrobium*).

The family at present contains three genera *Stramentimicrobium* gen. nov., *Oleincola* gen. nov. and *Oleihabitans* gen. nov.

Type genus: *Stramentimicrobium*.

**Description of *Pheonicimicrobiales* ord. nov. (O2)**

*Phoenicimicrobiales* (Phoe.ni.ci.mi.cro.bi.a’les. N.L. neut. n. *Phoenicimicrobium* type genus of the order; L. suff. –*ales* ending to denote an order; N.L. fem. pl. n. *Phoenicimicrobiales* the *Phoenicimicrobium* order).

The order *Phoenicimicrobiales* comprised of two families *Pheonecimicrobiaceae* fam. nov. and *Stramentimicrobiaceae* fam. nov.

Type genus: *Phoenicimicrobium*.

**Description of *Phoenicimicrobiia* class. nov. (C2)**

*Phoenicimicrobiia* (Phoe.ni.ci.mic.ro.bi’i.a. N.L. fem. pl. n. *Phoenicimicrobiales* type order of the class; L. suff. –*ia* ending to denote a class; N.L. neut. pl. n. *Phoenicimicrobia* the class of the order *Phoenicimicrobiales*).

The description of the class is the same as for the order *Phoenicimicrobiales*.

**References**

1. Delcher AL, Salzberg SL, Phillippy AM. Using MUMmer to identify similar regions in large sequence sets*.* Curr Protoc Bioinform. 2003;1:10-3.

2. Pritchard L, Glover RH, Humphris S, Elphinstone JG, Toth IK. Genomics and taxonomy in diagnostics for food security: soft-rotting enterobacterial plant pathogens. Anal. Methods. 2016;8:12-24.

3. Jiao JY, Fu L, Hua ZS, Liu L, Salam N, Liu PF, et al. Insight into the function and evolution of the Wood–Ljungdahl pathway in *Actinobacteria.* ISME J. 2021;15:3005-3018.

4. Jiao JY, Lian ZH, Li MM, Salam N, Zhou EM, Liu L, et al. Comparative genomic analysis of *Thermus* provides insights into the evolutionary history of an incomplete denitrification pathway*.* mLife. 2022;1:198-209.

5. Hua ZS, Wang YL, Evans PN, Qu YN, Goh KM, Rao YZ, et al. Insights into the ecological roles and evolution of methyl-coenzyme M reductase-containing hot spring Archaea*.* Nat Commun. 2019. 10(1):4574.

6. Greening C, Biswas A, Carere CR, Jackson CJ, Taylor MC, Stott MB, et al. Genomic and metagenomic surveys of hydrogenase distribution indicate H_2_ is a widely utilised energy source for microbial growth and survival*.* ISME J. 2016;10:761-777.

7. Edgar RC. MUSCLE: multiple sequence alignment with high accuracy and high throughput*.* Nucleic Acids Res. 2004;32:1792-1797.

8. Capella-Gutiérrez S, Silla-Martínez JM, Gabaldón T. trimAl: a tool for automated alignment trimming in large-scale phylogenetic analyses*.* Bioinformatics. 2009;25:1972-1973.

9. Nguyen LT, Schmidt HA, Von Haeseler A, Minh BQ. IQ-TREE: a fast and effective stochastic algorithm for estimating maximum-likelihood phylogenies*.* Mol Biol Evol. 2015;32:268-274.

10. Kalyaanamoorthy S, Minh BQ, Wong TK, Von Haeseler A, Jermiin LS. ModelFinder: fast model selection for accurate phylogenetic estimates*.* Nat Methods. 2017;14:587.

11. Matheus Carnevali PB, Schulz F, Castelle CJ, Kantor RS, Shih PM, Sharon I, et al. Hydrogen-based metabolism as an ancestral trait in lineages sibling to the *Cyanobacteria.* Nat Commun. 2019;10: 463.

12. Liu YF, Qi ZZ, Shou LB, Liu JF, Yang SZ, Gu JD, et al. Anaerobic hydrocarbon degradation in candidate phylum ‘*Atribacteria*’ (JS1) inferred from genomics. ISME J. 2019;13:2377-2390.

13. Adam PS, Borrel G, Gribaldo S. Evolutionary history of carbon monoxide dehydrogenase/acetyl-CoA synthase, one of the oldest enzymatic complexes*.* Proc. Natl Acad Sci USA. 2018;115:E1166-E1173.

14. Letunic I, Bork P. Interactive Tree Of Life (iTOL) v4: recent updates and new developments*.* Nucleic Acids Res. 2019;47:W256-W259.

15. Edgar RC. Muscle5: High-accuracy alignment ensembles enable unbiased assessments of sequence homology and phylogeny*.* Nat Commun. 2022;13:6968.

16. Katayama T, Nobu MK, Kusada H, Meng XY, Hosogi N, Uematsu K, et al. Isolation of a member of the candidate phylum ‘*Atribacteria*’ reveals a unique cell membrane structure*.* Nat Commun. 2020;11: 6381.

17. Chaumeil PA, Mussig AJ, Hugenholtz P, Parks DH. GTDB-Tk: a toolkit to classify genomes with the Genome Taxonomy Database. Bioinformatics. 2020;36:1925–1927.

18. Chun J, Oren A, Ventosa A, Christensen H, Arahal DR, da Costa MS, et al., Proposed minimal standards for the use of genome data for the taxonomy of prokaryotes. Int J Syst Evol. 2018;68:461-466.

19. Konstantinidis KT, Rosselló-Móra R, Amann R. Uncultivated microbes in need of their own taxonomy*.* ISME J. 2017;11:2399-2406.
